# Bacterial wilt affects the structure and assembly of microbial communities along the soil-root continuum

**DOI:** 10.1186/s40793-024-00548-7

**Published:** 2024-01-16

**Authors:** Jinchang Liang, Chengjian Wei, Xueru Song, Rui Wang, Heli Shi, Jun Tan, Dejie Cheng, Wenjing Wang, Xiaoqiang Wang

**Affiliations:** 1 Key Laboratory of Tobacco Pest Monitoring & Integrated Management, Tobacco Research Institute of Chinese Academy of Agricultural Sciences, 266101 Qingdao, China; 2 College of Agriculture, Guangxi University, 530004 Nanning, China; 3 Engineering Center for Biological Control of Diseases and Pests in Tobacco Industry, 653100 Yuxi, China; 4 Enshi Tobacco Science and Technology Center, 445000 Enshi, China

**Keywords:** Microbiome, Assembly mechanisms, Functional profile

## Abstract

**Background:**

Beneficial root-associated microbiomes play crucial roles in enhancing plant growth and suppressing pathogenic threats, and their application for defending against pathogens has garnered increasing attention. Nonetheless, the dynamics of microbiome assembly and defense mechanisms during pathogen invasion remain largely unknown. In this study, we aimed to investigate the diversity and assembly of microbial communities within four niches (bulk soils, rhizosphere, rhizoplane, and endosphere) under the influence of the bacterial plant pathogen *Ralstonia solanacearum*.

**Results:**

Our results revealed that healthy tobacco plants exhibited more diverse community compositions and more robust co-occurrence networks in root-associated niches compared to diseased tobacco plants. Stochastic processes (dispersal limitation and drift), rather than determinism, dominated the assembly processes, with a higher impact of drift observed in diseased plants than in healthy ones. Furthermore, during the invasion of *R. solanacearum*, the abundance of *Fusarium* genera, a known potential pathogen of *Fusarium* wilt, significantly increased in diseased plants. Moreover, the response strategies of the microbiomes to pathogens in diseased and healthy plants diverged. Diseased microbiomes recruited beneficial microbial taxa, such as *Streptomyces* and *Bacilli*, to mount defenses against pathogens, with an increased presence of microbial taxa negatively correlated with the pathogen. Conversely, the potential defense strategies varied across niches in healthy plants, with significant enrichments of functional genes related to biofilm formation in the rhizoplane and antibiotic biosynthesis in the endosphere.

**Conclusion:**

Our study revealed the varied community composition and assembly mechanism of microbial communities between healthy and diseased tobacco plants along the soil-root continuum, providing new insights into niche-specific defense mechanisms against pathogen invasions. These findings may underscore the potential utilization of different functional prebiotics to enhance plants’ ability to fend off pathogens.

**Supplementary Information:**

The online version contains supplementary material available at 10.1186/s40793-024-00548-7.

## Introduction

Plants harbor diverse microbial communities that interact with plants and evolve for mutual benefits [1]. Plant microbiomes, especially root-associated ones, improve plant fitness by impacting various aspects, including nutrient availability, growth promotion, abiotic stress tolerance, and pathogen suppression [2]. Based on the proximity to the root, the ecological niche can be categorized into three distinct regions: the rhizosphere, rhizoplane, and endosphere, each of which has been observed to recruit a diverse microbiome [2, 3]. Additionally, using root-associated beneficial microbiota for agricultural production is increasingly considered an alternative approach to promoting green and sustainable agricultural development [4]. Hence, understanding the ecological assembly mechanism and functional changes in microbiomes along the soil-root continuum under different health conditions is necessary to precisely use beneficial microbiomes in specific niches.

Microbial communities are distributed in all plant parts and act as the first line of defense against pathogen invasion [5]. Disruption of the balance of microbial communities around the root increases disease incidence, possibly due to the disproportionate growth of pathogens [6]. Microbial diversities and community compositions were reportedly associated with disease resistance. A higher diversity of microbial communities usually results in more complex and robust microbial co-occurrence networks, thereby enhancing the ability of plants to defend against disease invasion [7]. Additionally, some taxa recruited by plants can improve their resistance ability to disease in different ways. For instance, three bacterial species were specifically promoted by *Arabidopsis thaliana* in the rhizosphere to activate foliar defense against the invasion of the downy mildew pathogen *Hyaloperonospora arabidopsis* [8]. Root exudation of sucrose enhances the colonization of *Bacillus subtilis* on the roots to defend against *Fusarium* wilt in tomatoes [9]. Moreover, some plants use the “cry for help” strategy to recruit beneficial microbes to resist biotic and abiotic stresses [10]. For example, with the invasion of pathogenic *Fusarium pseudograminearum*, *Stenotrophomonas* was accumulated in the root-associated niches of wheat, and re-introducing the bacterial strain to rhizosphere soils enhanced the ability of plants to suppress disease [10]. Additionally, some antibiotic-producing bacteria, including *Streptomyces*, *Bosea*, and *Pseudomonas*, were enriched in diseased tobacco with bacterial wilt, which may act as ‘pathogen antagonists’ to pathogenic *R. solanacearum* [11]. Although several studies have researched the resistance mechanisms of beneficial microorganisms against plant diseases, a systematic investigation of the defense strategies of microbiomes in different ecological niches is still lacking.

Bacterial wilt diseases caused by *R. solanacearum* could infect many Solanaceae crops, including eggplant, pepper, and tobacco, and cause devastating losses in crop production worldwide [12]. *R. solanacearum* can exist in the soil for several years, posing a continuous threat to plant health [13]. In recent decades, the incidence of bacterial wilt has increased due to long-term continuous monoculture cropping [14]. Previous studies have reported distinct community compositions of microorganisms between healthy and diseased plants, although they share similar environmental conditions [11, 14, 15]. Additionally, bacterial wilt significantly decreased microbial community diversity, resulting in reduced complexity and robustness of microbial networks [13]. Therefore, specific bacterial groups were hypothesized to be present in root-associated niches of healthy plants to maintain the balance of microbial communities and help plants suppress disease occurrence. Nevertheless, most previous research targeted rhizosphere microorganisms, and the assembly mechanisms and indicator taxa of microbiomes that respond to bacterial wilt invasion in different niches remain unclear.

The assembly process and community structure of rhizosphere microbial communities were significantly influenced by niches [16, 17], therefore, we hypothesized that the response of microbiome to pathogen invasion varies along soil-root continuums. Furthermore, given that the evidence linking microbial taxonomy and community functions [16, 18, 19], we hypothesized that the ways by which beneficial microorganisms inhibit plant pathogens vary depending on the ecological niches and plant health conditions. To test these hypotheses, changes in microbial abundance and community composition during pathogen invasion were investigated using qPCR and high-throughput amplicon sequencing. In addition, indicator taxa for resistance to bacterial wilt and maintenance of community stability were identified using microbial network analysis. Moreover, microbial functional profiles in different niches were predicted, and variances in microbial functions between healthy and diseased tobacco plants were compared. This study provides a detailed assessment of the response of microbial communities to pathogen invasion in different niches and will thus increase our understanding of microbial assembly mechanisms and the varied defense strategies of plants under pathogen stress.

## Materials and methods

### Sample selection and collection

Samples were collected from tobacco fields cropped continuously for decades in Xinping (23°58′56″ N, 101°42′46″ E), Yunnan Province in China on August 2, 2022. Tobacco (variety Yunxue 39) and rice are rotated on the sampled fields. For the diseased samples, tobacco plants with lightly infected symptoms (grade 1 infection) of bacterial wilt were selected [20], which was subsequently confirmed by quantifying the absolute abundance of *R. Solanacearum*. Healthy samples were obtained from plots with no evidence of bacterial wilt. The healthy plots were close to the diseased plots to avoid deviations caused by geographical factors. Six replicates of soil and root samples were collected from healthy and diseased fields.

Bulk soils, rhizosphere, rhizoplane, and endosphere compartments were sampled as described by Edwards et al. [3], with some modifications. Specifically, the top 1 cm soils were removed, and the bulk soils (1 ~ 15 cm depth) were sampled at a distance of 20 cm from the roots before uprooting the tobacco plants from the soil. The loosely attached soils were shaken off roots, leaving about 2 mm of soil attached. The remaining soils were sampled by gently brushing the soils on the roots and were regarded as the rhizosphere samples. To sample the rhizoplane samples, the remaining root sections were placed in a sterile flask containing 50 mL of sterile phosphate-buffered saline, stirred vigorously, and sonicated twice (40 kHz for 1 min) to detach the microbes adhering to the root surface. Next, the solutions were centrifuged (7,000 × g, 10 min, and 4 °C), and the resultant pellets were rhizoplane samples. Furthermore, the sonicated roots were soaked in alcohol (75%) for 5 min and sodium hypochlorite (5%) for 5 min. Afterward, the root sections were rinsed thrice in sterile water, and the microbial community remaining in the roots was considered as endosphere microbiomes. All the samples were stored at -80 °C until use. In total, 48 samples (2 health conditions × 6 replicated × 4 compartments) were collected, including healthy and diseased samples, with four compartments.

### DNA extraction and microbial abundance quantification

The total DNA was extracted from all the samples using a DNeasy® PowerSoil® Kit (QIAGEN, Germany). DNA quantity and quality were evaluated using a Nanodrop-2000 spectrophotometer (Thermo Fisher Scientific Corp., Waltham, MA, USA), and the high-quality DNA was stored at -80 °C until use.

The abundances of total bacteria, fungi, *R. solanacearum*, and *Fusarium* sp. were quantified using qPCR and the QuantStudio3 real-time system (Thermo Fisher). The primers set A-967F (5’-CAACGCGAAGAACCTTACC-3’) /B-1046R (5’-CGACAGCCATGCANCACCT-3’) [21] and ITS1f (5’-TCCGTAGGTGAACCTGCGG-3’) /5.8s (5’-CGCTGCGTTCTTCATCG-3’) [22] were used to quantify the bacterial and fungal abundances. Additionally, Rsol_fliCf (5’-GAACGCCAACGGTGCGAACT-3’) /Rsol_fliCr (5’-GGCGGCCTTCAGGGAGGTC-3’) primers targeting the *fliC* gene [23] and FusEF (5’-CTGGGTTCTTGACAAGCTCA-3’)/FusER (5’-CGGTGACATAGTAGCGAGGA-3’) primer targeting the *EF1α* gene [24] were used to quantify the abundance of *R. solanacearum* and *Fusarium* genus, respectively. Each 20-µL real-time PCR contained 10 µL of SYBR Green Real-time PCR Master Mix (TaKaRa, Tokyo, Japan), 0.4 µL of each primer (10 µM), 2 µL of 10 times diluted DNA, and double-distilled water added up to 20 µL. The PCR conditions were as described previously [21–24]. Clones with the bacterial 16S rRNA gene (for bacteria), ITS gene (for fungi), *fliC* gene (for *R. solanacearum*), and *EF1α* gene (for *Fusarium* sp.) were used to construct a standard curve. The qPCR amplification efficiency was between 96% and 103% with an R^2^ value of 0.99.

### Illumina sequencing and data analysis

To identify the microbial community composition, the hypervariable V5-V7 region in the 16S rRNA gene and ITS region 1 of the nuclear ribosomal coding cistron was amplified from the DNA samples using primers 799F (5’- AACMGGATTAGATACCCKG-3’) /1193R (5’- ACGTCATCCCCACCTTCC − 3’) [25, 26] and ITS1F (5’-CTTGGTCATTTAGAGGAAGTAA-3’) /ITS2R (5’-GCTGCGTTCTTCATCGATGC-3’) [27]. Primers 799 F/1193R were widely used in amplicon sequencing of endophytic microbiome, due to yielding low co-amplification levels of chloroplast and mitochondrial genes. [28]. The PCR reactions were performed in a 50-µL reaction solution containing 10 µL 5 × FastPfu Buffer, 5 µL 2.5 mM dNTPs, 1 µL of both 10 µM primers, 1 µL FastPfu polymerase, 0.5 µL bovine serum albumin (BSA) and 10 ng template DNA. The PCR conditions were as previously described [25–27]. The PCR products were purified using the AxyPrep DNA Gel Extraction Kit (Axygen Biosciences, Union City, CA, USA). Qualified PCR products were sequenced on an Illumina MiSeq platform (Illumina, San Diego, CA, USA) at Magigene Technology Co., Ltd. (Guangzhou, China).

Raw reads were processed using Quantitative Insights into Microbial Ecology II (QIIME II) [29]. After cutting the primer sequences and filtering low-quality read ends with quality scores below 20, DADA2 was used to denoise and merge the high-quality reads and then split them into amplicon sequence variants (ASVs). The 16S rRNA and ITS sequences were taxonomically assigned by comparison with SILVA (SSU138) [30] and UNITE (v8.3) databases [31], respectively. The ASVs from 16S rRNA amplicons annotated as chloroplasts or mitochondria that could not be annotated at the boundary level were removed for further analysis. Sequences in all samples were rarefied according to the minimum number of sequences. Finally, 62,660 sequences for bacteria and 90,474 sequences for fungi remained in each sample for downstream analyses.

### Statistical analysis

Alpha diversity indices, including Chao 1, Shannon, Simpson, ACE index, and Good’s coverage, were calculated to estimate the diversity of the community using normalized reads. Variances in alpha diversity between different groups were statistically compared using the t-test in the GraphPad Prism version 8 (GraphPad Software, San Diego, CA, USA). For β-diversity analysis, PERMANOVA was used to assess the differences between different groups for the bacterial and fungal communities based on the Bray–Curtis distance, and NMDS was used for data visualization. The above analyses were conducted using the Vegan package of the R software (v3.5.3). Differences in absolute abundance across samples were evaluated using the t-test performed in GraphPad Prism v8.

To determine the relative contributions of determinism and stochasticity in the ecological process, R “picante” library was used to calculate Beta Nearest Taxon Index (βNTI). βNTI values > 2 or < − 2 represent a deterministic process, indicating heterogeneous and homogeneous selection, respectively [32].|βNTI| < 2 indicates stochastic processes, including homogenizing dispersal [Bray–Curtis-based Raup–Crick matrix (RCbray) < -0.95], dispersal limitation (RCbray > 0.95), and drift (|RCbray| < 0.95) [32]. Additionally, the SourceTracker model was used to identify the effects of different microorganisms to the enrichment process from the bulk soils to the endosphere. The percentage values were counted with the functions “sourcetracker” and “predict” packages in R [33]. Moreover, to identify the enriched ASVs in diseased tobacco plants, the R package “edgeR” was used to perform the differential enrichment analysis of microbial ASVs’ abundance (relative abundance > 0.01%). Only the ASVs with *P* < 0.05 (FDR-adjusted) and log_2_ (fold change) > 1 or < − 1 were regarded as “enriched” or “depleted,” respectively. Venn diagrams across different compartments were obtained using Venn software [34]. Bar charts were visualized using OriginPro (v2023, OriginLab Corporation, Northampton, MA, USA).

Co-occurrence networks for bacterial and fungal communities in different groups were constructed using a network analysis pipeline (Molecular Ecological Network Analysis Pipeline, http://ieg2.ou.edu/MENA). To reduce noise and thus reduce false-positive predictions, genera (relative abundance > 0.01%) present in more than one-third of the samples were included in the network analysis. Additionally, Spearman’s correlation coefficients were used to calculate the correlations among different species. Networks were constructed using methods based on random matrix theory [35, 36]. Nodes with high degrees (top 20%) and low betweenness centrality (ranking in the bottom 20% of nodes with high degrees) were defined as keystone species in each network [37]. Lastly, network visualization was conducted using the Gephi (v0.9.2) [38].

To characterize the differences in microbial potential functions, functional profiles were predicted using PICRUSt2 [39]. Chao 1 index was calculated to estimate the diversity functions using normalized data. Differences in the functional compositions of different groups were assessed using PERMANOVA, and NMDS was used for data visualization. Finally, statistical analysis of metagenomic profiles was used to calculate the functions that mainly contributed to the differences in community structure across groups [40].

## Results

### Abundance of bacterial and fungal community

**Fig. 1:**
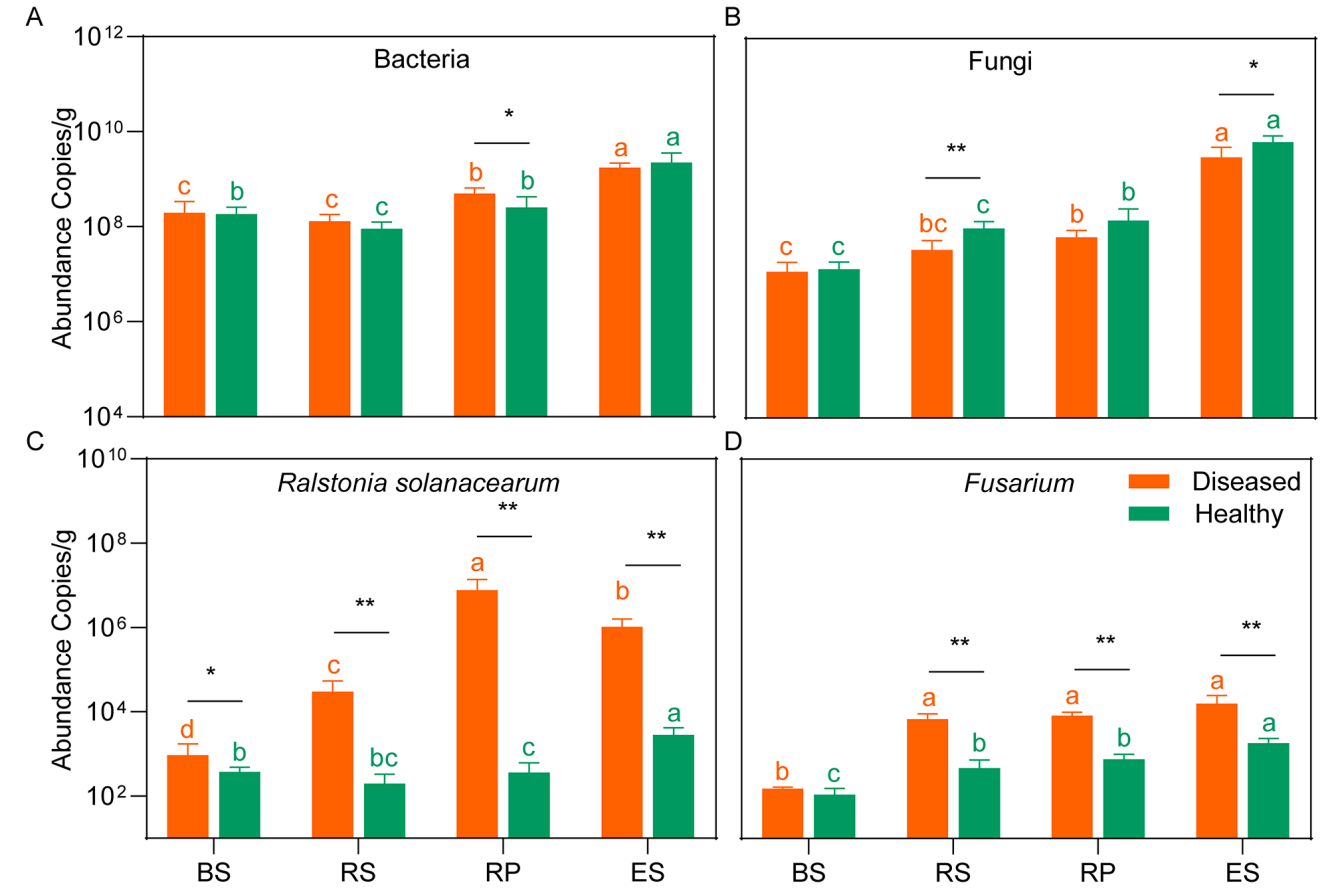
Bacterial and fungal abundance derived from qPCR based on the 16S rRNA **(A)**, ITS **(B)**, *flic*  **(C)**, and *EF1α* gene **(D)**. The asterisks (*) indicate significant differences between diseased and healthy tobacco within the same niche. *, *P* < 0.05, **, *P* < 0.01, or ***, *P* < 0.001. Lowercase letters over the boxplot indicate statistically significant differences (t-test, *P* < 0.05) across niches in healthy or diseased plants. BS: bulk soils; RS: rhizosphere; RP: rhizoplane; ES: endosphere

The bacterial 16S rRNA gene abundance varied from 9.10 ± 3.3 × 10^7^ copies g^− 1^ (in rhizosphere soils of healthy tobacco plants) to 9.10 ± 1.3 × 10^9^ copies g^− 1^ (in the endosphere of healthy tobacco plants) across all the samples, with a significantly higher abundance in endosphere and rhizoplane than in bulk and rhizosphere soils (*P* < 0.05, t-test). Additionally, no significant variances in the bacterial abundance were found between diseased and healthy tobacco plants, except for samples derived from the rhizoplane (Fig. 1A). The fungal ITS gene copy showed an increased trend from the bulk soils to endosphere, with the lowest abundance in the bulk soils of diseased tobacco plants (1.21 ± 0.70 × 10^7^ copies g^− 1^) and highest abundance in endosphere of diseased tobacco plants (6.57 ± 2.40 × 10^9^ copies g^− 1^). Moreover, the fungal abundance was higher in healthy tobacco plants than in diseased tobacco plants in rhizosphere and endosphere niches (*P* < 0.05, t-test, Fig. 1B). The absolute abundance of *R. solanacearum* and *Fusarium* sp. were identified using *flic* gene and *EF1α* gene, respectively. As expected, significantly higher abundances of *R. solanacearum* and *Fusarium* were observed in diseased tobacco plants than in healthy tobacco plants of rhizosphere, rhizoplane, and endosphere niches (*P* < 0.05, Fig. 1C and D). Additionally, the highest abundance of *R. solanacearum* was quantified in the rhizoplane of diseased tobacco plants (7.62 ± 0.60 × 10^6^ copies g^− 1^, Fig. 1C). Compared with healthy tobacco plants, *R. solanacearum* densities in diseased tobacco plants increased more sharply in the rhizoplane and rhizosphere (approximately 21,013 and 364 folds, respectively), suggesting that invasions by pathogens might have serious effects on the niches close to the root. Furthermore, the gene copies of *Fusarium* varied from 1.10 ± 0.44 × 10^2^ to 1.59 ± 0.87 × 10^4^ copies g^− 1^ across all the samples and were lower in bulk soils than any other niches (*P* < 0.05, Fig. 1D).

### Bacterial and fungal diversity and community assembly

**Fig. 2:**
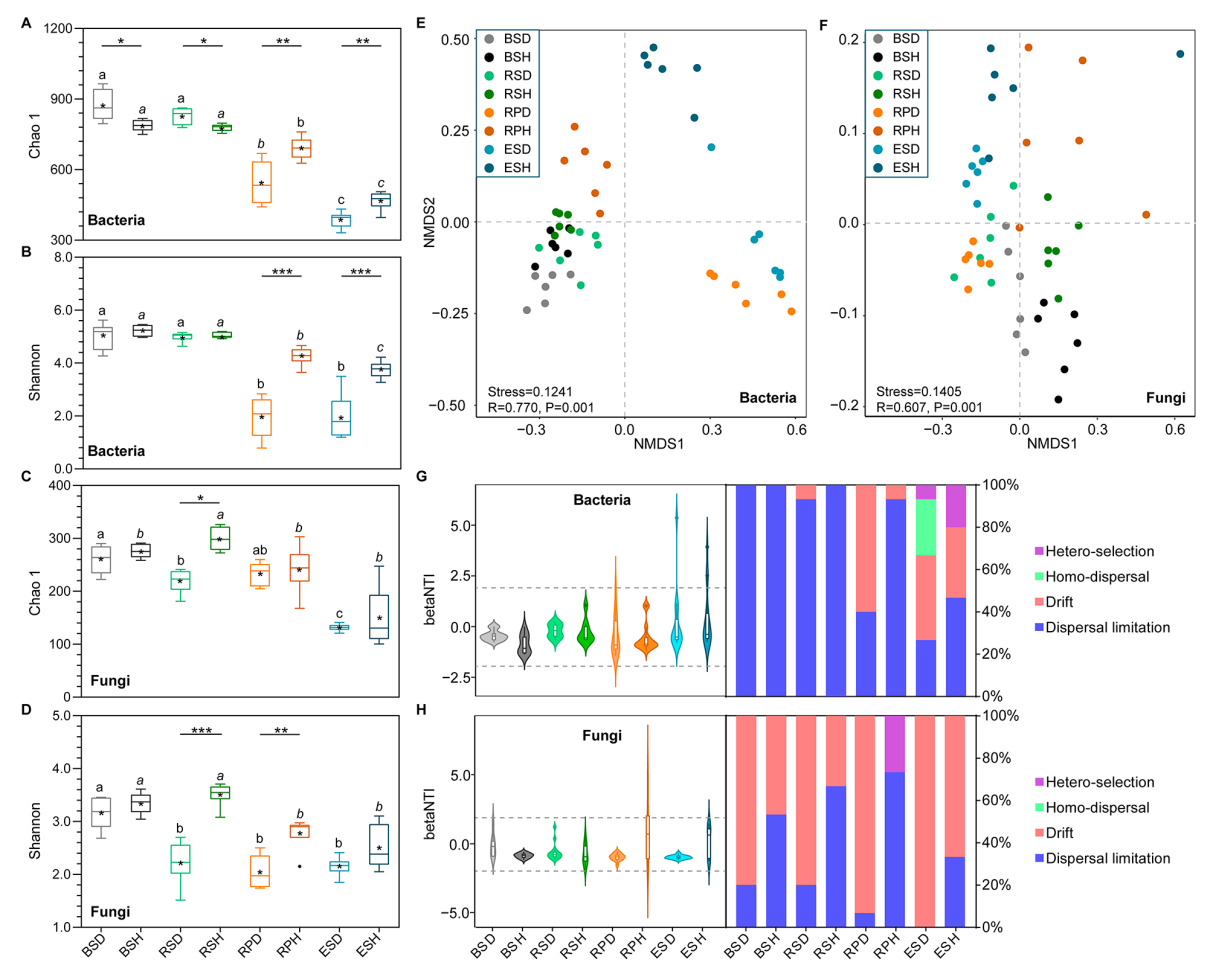
Alpha diversity and assembly of microbial communities. **(A)** Bacterial Chao 1, **(B)** bacterial Shannon, **(C)** fungal Chao 1, and **(D)** fungal Shannon indices. Stars within the boxplot define the mean value, and outliers are plotted as circles if present. BS, bulk soils; RS, rhizosphere; RP, rhizoplane; ES, endosphere; D, diseased tobacco plants; H, healthy tobacco plants. The asterisks (*) indicate significant differences between healthy and diseased tobacco plants within same niche. *, *P* < 0.05, **, *P* < 0.01, or ***, *P* < 0.001. Lowercase letters over the boxplot indicate statistically significant differences (t-test, *P <* 0.05) across niches within diseased (letters in regular) or healthy groups (letters in italics). NMDS plot showing the ordination of bacterial **(E)** and fungal **(F)** communities among all samples. Samples in different groups are color-coded. Deterministic and stochastic processes in bacterial **(G)** and fungal **(H)** communities of diseased and healthy tobacco plants among four niches

A total of 5,143,167 and 8,310,002 sequences were left after merging and filtering 16S rRNA and ITS raw reads from the 48 samples, respectively. These sequences were clustered into 15,313 and 5,897 amplicon sequence variants (ASVs) for 16S rRNA and ITS data, respectively. Additionally, Good’s coverage values were > 99.83% across samples for 16S and ITS data (Table S1), indicating that sequencing depths recovered almost all diversity of the local species in these samples. For the bacterial community, the Chao 1 and Shannon indices were higher in the bulk and rhizosphere soils of diseased tobacco plants than healthy tobacco plants; conversely, these indices were dramatically higher in the rhizoplane and endosphere of healthy tobacco plants than diseased tobacco plants (Fig. 2A and B, *P* < 0.05). Additionally, the Chao 1 and Shannon indices for the fungal communities were higher in healthy tobacco plants than in diseased tobacco plants in the rhizosphere and rhizoplane soils (Fig. 2C and D, *P* < 0.05). Bacterial communities in bulk and rhizosphere soils had higher alpha diversity indices than the rhizoplane soils and the root endosphere (Fig. 2, *P* < 0.05). Moreover, fungal community diversity in the endosphere was markedly lower than that in the bulk soils (Fig. 2, *P* < 0.05).

**Table 1:** R^2^ and *P* values calculated by PERMANOVA for the variance of bacterial and fungal communities among different samples

Factor(s)	Results by factor			Results by health condition/niche		
	R^2^	*P* value		Comparison	R^2^	*P* value
Health condition (Bacteria)	0.022	0.343		BSD vs. BSH	0.183	**0.004**
				RSD vs. RSH	0.342	**0.001**
				RPD vs. RPH	0.638	**0.004**
				ESD vs. ESH	0.517	**0.003**
Niche (Bacteria)	0.718	**0.001**		BSD vs. RSD vs. RPD vs. ESD	0.734	**0.001**
				BSH vs. RSH vs. RPH vs. ESH	0.583	**0.001**
Health condition & niche (Bacteria)	0.448	**0.001**				
Health condition (Fungi)	0.079	**0.003**		BSD vsBSH	0.416	**0.004**
				RSD vsRSH	0.488	**0.005**
				RPD vsRPH	0.338	**0.001**
				ESD vsESH	0.224	**0.002**
Niche (Fungi)	0.511	**0.001**		BSD vs. RSD vs. RPD vs. ESD	0.503	**0.001**
				BSH vs. RSH vs. RPH vs. ESH	0.318	**0.001**
Health condition & niche (Fungi)	0.217	**0.001**				

*P* values in bold indicate significant correlations (*P* < 0.05; n = 6). BS: bulk soils; RS: rhizosphere; RP: rhizoplane; ES: endosphere; D: diseased tobacco plants; H: healthy tobacco plants

Non-metric multidimensional scaling (NMDS) and permutational multivariate analysis of variance (PERMANOVA) of bacterial and fungal data showed that variations in microbial communities were influenced by both diseases and niches. NMDS analysis revealed that all samples were partitioned into eight groups based on health conditions and niches of the microbial communities (Fig. 2E and F). Additionally, PERMANOVA showed that differences in the microbial community were mainly due to compartment niches (71.8% for bacteria, *P* < 0.01, and 51.1% for fungi, *P* < 0.01) and disease (2.2% for bacteria, and 7.9% for fungi, *P* < 0.01, Table 1)). Marked variances in microbial communities were found between diseased and healthy tobacco plants (R^2^ = 18.3–63.8%, *P* < 0.01, Table 1). Furthermore, the compartment niches showed significant variations in microbial communities in both healthy and diseased tobacco plants (R^2^ = 31.8–73.4%, *P* < 0.001, Table 1).

Neutral processes were inferred to identify community assembly mechanisms. Dispersal limitation was the stochastic process driving bacterial community assembly, accounting for 75.0% on average across all samples (Fig. 2G). This was followed by drift, especially in the rhizoplane (33.3% on average) and the endosphere (33.67% on average). A more important role of dispersal limitation for bacterial community assembly was shown in healthy tobacco plants than in diseased tobacco plants. Furthermore, dispersal limitation and drift were important for fungal community assembly, and their influence significantly differed between diseased and healthy tobacco plants. Drift exerted a more notable influence on the assembly of fungal communities in diseased tobacco plants (approximately 88.3%) than in healthy tobacco plants (45.0%); however, dispersal limitation was more important in healthy tobacco plants (56.7%) than in diseased tobacco plants (approximately 11.7%). These results suggest that stochastic processes contribute more to variations in bacterial and fungal communities than deterministic processes.

The SourceTracker model showed that microbial communities mainly originated from bulk soils; however, the trends differed between diseased and healthy samples (Fig. S1). The bacterial communities of bulk soils were the major sources of rhizosphere microbiomes for diseased samples, with a contribution of 80.3%; however, in healthy samples, majority of species in the rhizosphere were sourced from the rhizoplane (60.2%) and less from the bulk soil (22.7%). Additionally, the major source of rhizoplane microbiomes in the diseased samples was the endosphere; however, the rhizoplane species (64.7%) were primarily derived from the rhizosphere in the healthy samples. Most of the endosphere species were originated from the rhizoplane in the diseased (83.3%) and healthy samples (39.3%); additionally, the rhizosphere soil was an important source of bacterial species (38.8%) in endosphere of healthy samples. For fungal communities, the majority of the rhizosphere species (67.0% for diseased samples and 58.3% for healthy samples) originated from bulk soils, and the fungal communities were gradually filtered at the rhizoplane and endosphere niches of the diseased samples. Lastly, the rhizoplane was the main potential source of fungal members in endosphere of diseased and healthy tobacco plants (Fig. S1).

### Changes in bacterial and fungal community composition

**Fig. 3:**
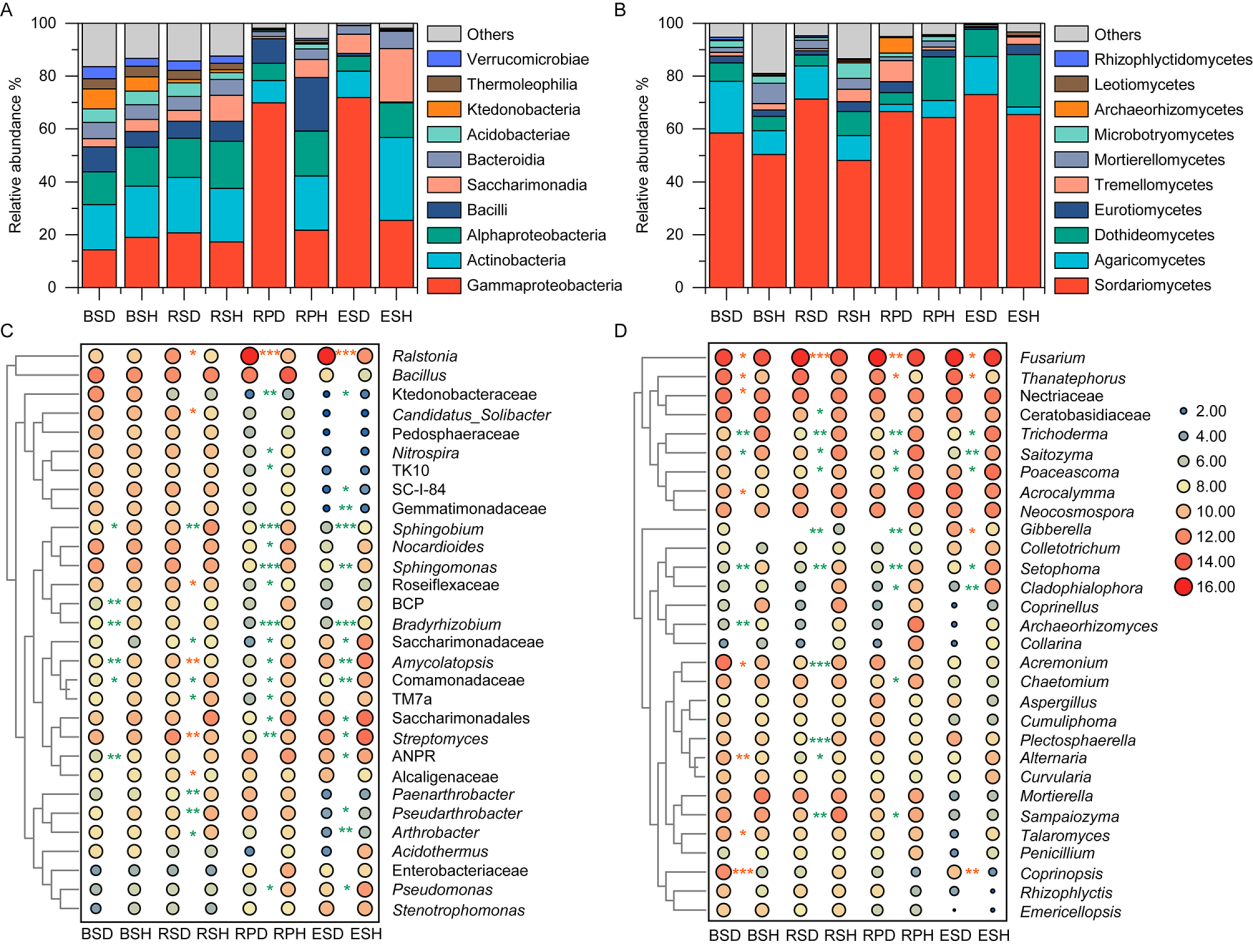
Community composition of the bacterial and fungal groups at the order **(A, B)** and genus (if not annotated to specific genera, using higher-level taxa to represent) levels **(C, D)**. The asterisks (*) in orange or green indicate a significantly higher relative abundance in diseased tobacco plants or healthy tobacco plants, respectively. *, *P* < 0.05, **, *P* < 0.01, or ***, *P* < 0.001. BCP, *Burkholderia*-*Caballeronia*-*Paraburkholderia*; ANPR, *Allorhizobium*-*Neorhizobium*-*Pararhizobium*-*Rhizobium*. BS: bulk soils; RS: rhizosphere; RP: rhizoplane; ES: endosphere; D: diseased tobacco plants; H: healthy tobacco plants

Taxonomic assignment revealed distinct variances at the class and genus levels across compartment niches (Fig. 3). In general, the number of bacterial and fungal taxa declined gradually from the bulk soils to the endosphere in all samples (Fig. S2). Regarding the bacterial community in diseased and healthy tobacco plants, the relative abundances of Gammaproteobacteria (47.2 ± 27.4%), and Saccharimonadia (8.8 ± 8.1%) were higher in the rhizoplane and endosphere; however, bulk and rhizosphere soils had higher proportions of Actinobacteria (19.4 ± 1.7%), Alphaproteobacteria (14.9 ± 2.2%), and Bacteroidia (5.8 ± 0.4%; Fig. 3A). Additionally, Saccharimonadia and Alphaproteobacteria were higher in healthy tobacco plants than in diseased tobacco plants in each compartment (*P* < 0.05). The most dominant classes in the fungal community were Sordariomycetes (62.2 ± 9.2%), Agaricomycetes (9.63 ± 5.8%), and Dothideomycetes (9.57 ± 5.8%), with varied relative abundances among the samples. Moreover, the bulk and rhizosphere soils had a greater percentage of Dothideomycetes than the rhizoplane and endosphere (12.8 ± 6.8% vs. 6.3 ± 2.2%); an opposite trend was observed for Agaricomycetes (6.6 ± 5.5% vs. 12.7 ± 4.9%; Fig. 3B). Furthermore, Sordariomycetes was dramatically enriched in diseased tobacco plants in each compartment (*P* < 0.05, t-test).

Notably, variances in microbial community compositions were more significant at the genus level. The bacterial genera (*Ralstonia*, *Bacillus*, and *Streptomyces*) and the fungal genera (*Fusarium*, *Trichoderma*, and *Thanatephorus*) were the dominant groups for the complete data, with variances in relative abundance across different compartments (Fig. 3C and D). Additionally, some bacterial genera, including *Nitrospira*, *Sphingobium*, *Nocardioides*, and *Sphingomonas* were enriched in the bulk and rhizosphere soils than in the other two niches. Additionally, indicator genera, including *Ralstonia*, *Pseudomonas*, and *Allorhizobium-Neorhizobium-Pararhizobium-Rhizobium* were more abundant in the rhizoplane and endosphere than in the other two compartments (Fig. 3C). Fungal genera, such as *Acrocalymma*, *Poaceascoma*, *Fusarium*, and *Neocosmospora* were enriched in the rhizoplane and endosphere than in the bulk and rhizosphere niches, while *Sampaiozyma*, *Mortierella*, and *Coprinellus* were significantly less abundant (Fig. 3D). Moreover, significant variances in the bacterial and fungal community were observed between diseased and healthy tobacco plants. Consistent with the quantitative PCR (qPCR) results, the relative abundances of *Ralstonia* and *Fusarium* were significantly higher in diseased tobacco plants (*P* < 0.05, t-test, Fig. 1C and D). The relative abundance of *Ralstonia* in diseased tobacco plants accounted for 1.26–63.87% of the total bacterial community composition, which was much higher in diseased tobacco plants than that in healthy tobacco plants (0.49–5.87%). Similarly, the average *Fusarium* relative abundance was higher in diseased tobacco plants (49.20%) than in healthy plants (25.90%). Compared with the bulk soil (six genera), the variances were more obvious (> 13 genera) in the other three compartments. Ten genera were enriched in the rhizoplane and endosphere of healthy tobacco plants, including *Sphingobium*, *Bradyrhizobium*, *Streptomyces*, and *Pseudomonas*, amongst others (Fig. 3C). Lastly, the fungal genera *Trichoderma*, *Saitozyma*, and *Setophoma* were significantly less abundant in diseased tobacco plants than in healthy tobacco plants (Fig. 3D).

**Fig. 4:**
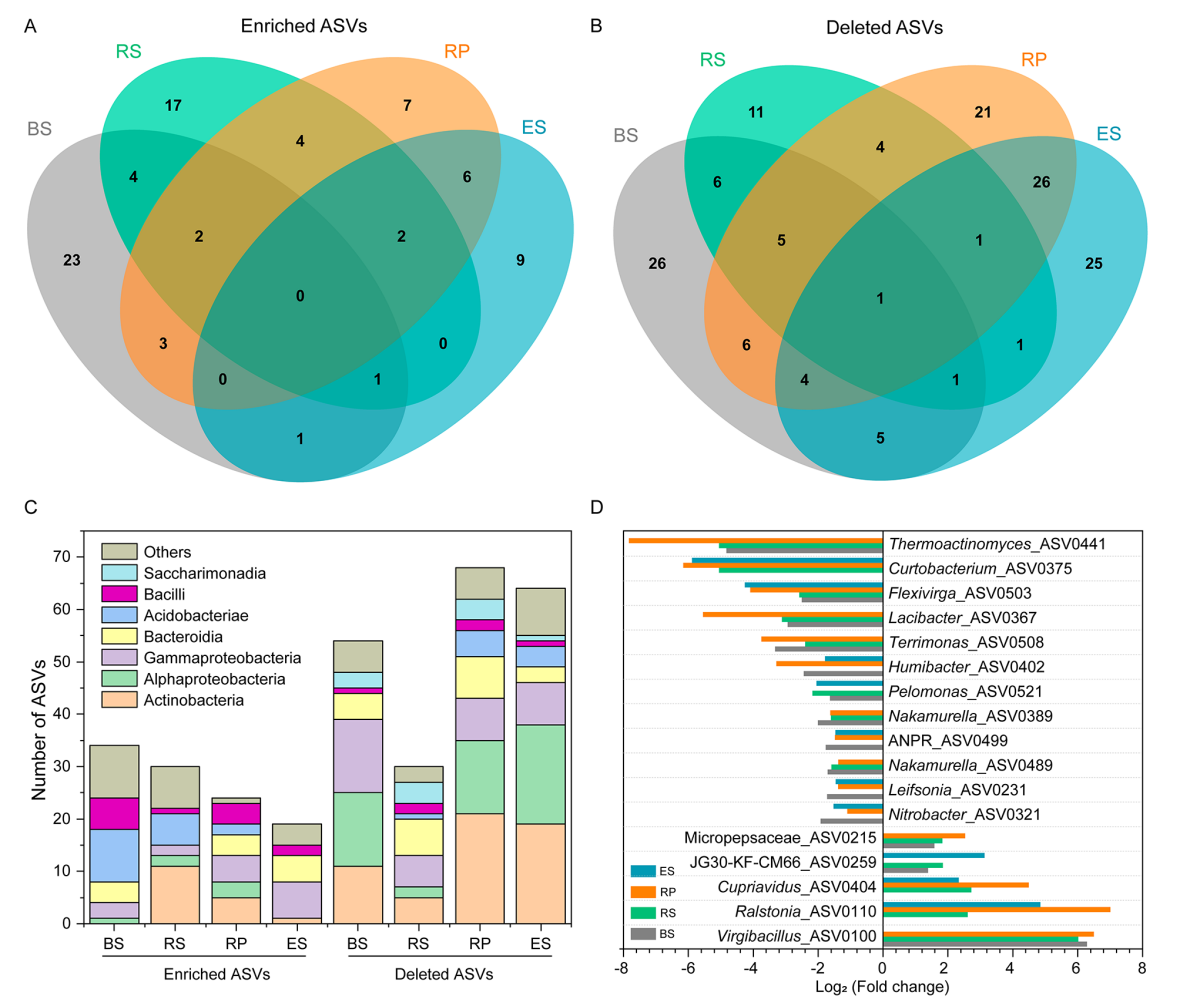
Taxonomic characteristics of differential bacteria between healthy and diseased tobacco plants. Venn diagram showing enriched **(A)** or depleted **(B)** ASVs’ number in each niche of diseased tobacco plants. The stacked bar chart indicates the taxonomy of enriched and deleted ASVs at the class level **(C)**. **(D)** Shared ASVs across diseased bulk soils, rhizosphere, rhizoplane, and endosphere at the genus level (If no specific genus is annotated, a higher-level taxonomic group is used to represent). BS: bulk soils; RS: rhizosphere; RP: rhizoplane; ES: endosphere

The enriched and deleted ASVs (relative abundance > 0.01% of the total sequences) in four compartments of diseased tobacco plants were calculated to identify the marker microbes beneficial to the tobacco plants (Fig. 4 and S3). The enriched numbers of bacterial ASVs in the rhizoplane (24) and endosphere (19) of diseased samples were slightly lower than those in bulk (34) and rhizosphere soils (30), while deleted ASVs showed the opposite trend (Fig. 4A and B). Specifically, the enriched ASVs were mainly classified as Acidobacteriae and Bacilli, and the deleted ASVs in diseased tobacco plants belonged to Actinobacteria, Alphaproteobacteria, Gammaproteobacteria, and Bacteroidia (Fig. 4C). Additionally, one deleted ASVs was shared by all the compartments, and 11 ASVs were shared by at least three compartments (Fig. 4C). Common ASVs belonged to different genera including *Curtobacterium*, *Lacibacte*, *Flexivirga*, and *Allorhizobium-Neorhizobium-Pararhizobium-Rhizobiumr*. (Fig. 4D). Moreover, the number of deleted fungal ASVs were much more than the enriched ASVs, both of which mainly belonged to Sordariomycetes, Dothideomycetes, and Eurotiomycetes (Fig. S4). Finally, none of the ASVs were shared by the four compartments, and two deleted ASVs (Pleosporales_ASV0061 and *Acremonium*_ASV0135) and one enriched ASV (*Umbelopsis*_ASV0110) were shared by all three compartments (Fig. S4D).

### Co-occurrence networks of diseased and healthy tobacco plants

**Fig. 5:**
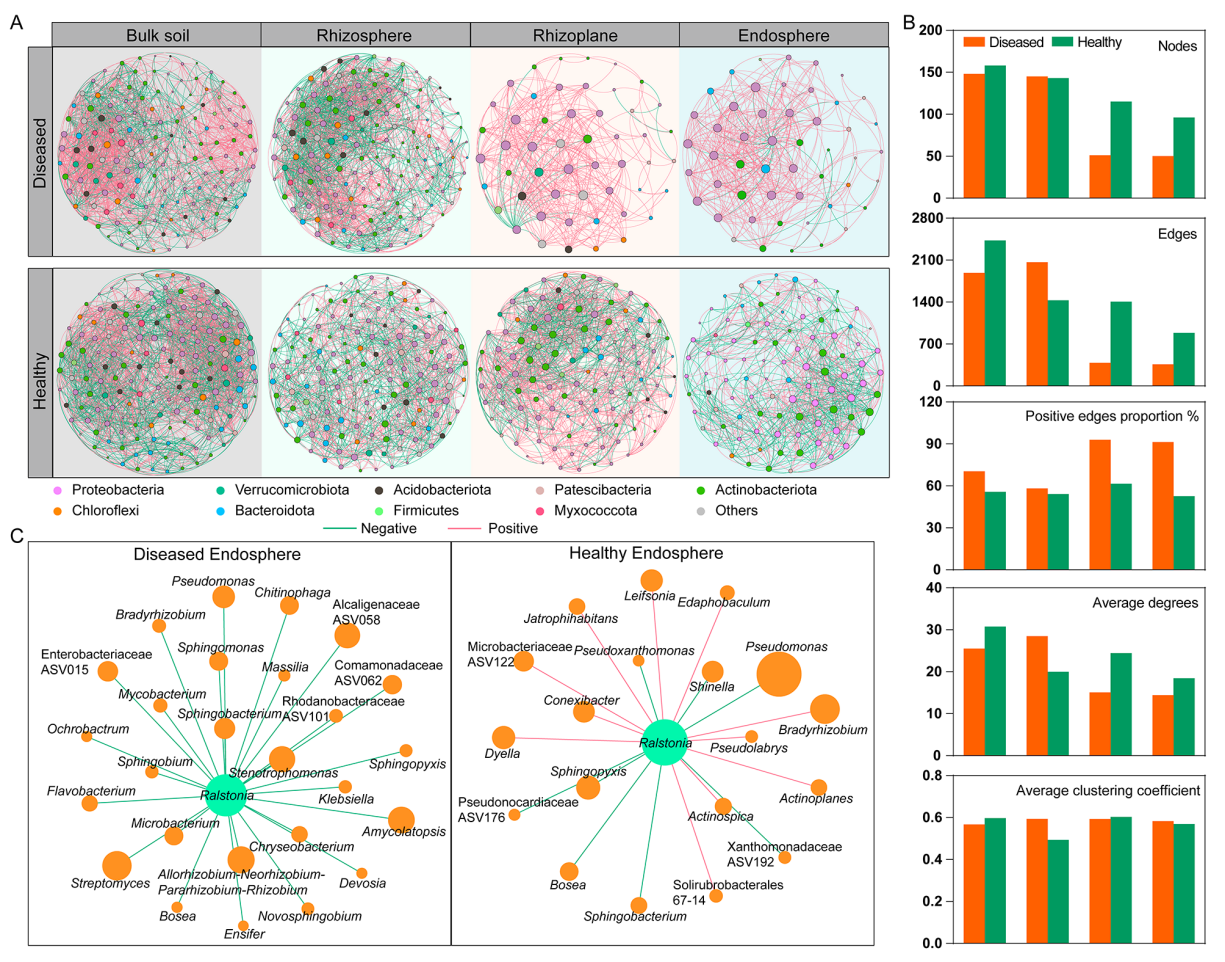
Pattens of the bacterial co-occurrence networks between diseased and healthy tobacco plants across four compartments. **(A)** Co-occurrence network analysis of diseased and healthy tobacco plants along the soil-root continuums. Nodes represent the dominant bacterial genera that are shown in different colors based on class taxonomy. The size of each node is proportional to the degree of the genus. Lines in red and green indicate positive and negative correlations, respectively. **(B)** Network topological parameters for bacterial networks in four niches. **(C)** The correlations between *Ralstonia* and other genera in diseased and healthy endosphere

To characterize the effect of disease on microbial interactions, co-occurrence patterns of bacterial and fungal communities in four niches were constructed (Fig. 5 and S5). Consistent with bacterial diversity (Fig. 2A), network complexity was higher in bulk soils and rhizospheres than in the other niches, with the highest average degree for networks in the diseased rhizosphere (28.469) and healthy bulk soil (30.734), and the lowest average degree in diseased (14.400) and healthy endosphere (18.458) (Fig. 5A and B, Table S2). The number of nodes and edges of the networks decreased significantly from the rhizosphere to the endosphere, indicating a strong influence of niches on bacterial networks (Fig. 5B). Additionally, the network complexity was higher in healthy samples than in diseased samples, with more nodes, edges, and a higher average degree in the three compartments of healthy tobacco plants (Fig. 5B). Moreover, the positive correlation varied slightly across the four compartments in healthy tobacco plants (52.6–61.5%); however, the positive correlation increased markedly from bulk soil and rhizosphere to root-associated niches in diseased tobacco plants (up to 93.0%, Fig. 5B and Table S2). Additionally, we constructed the co-occurrence networks using the dominant bacterial and fungal genera, and the patterns were similar to those of the bacterial networks (Fig. S5).

Based on the node degree (top 20%) and betweenness centrality (lower 20%), 63 keystone genera were identified, including 50 bacterial and 13 fungal genera, which may play crucial roles in maintaining the community structure and function of the microbiomes and defending against disease. Most of the keystone genera belonged to bacterial classes, including Gammaproteobacteria (15), Alphaproteobacteria (9), Acidobacteria (5), and the fungal class Sordariomycetes (8, Table S3). Additionally, some taxa, including *Allorhizobium*-*Neorhizobium*-*Pararhizobium*-*Rhizobium*, *Conexibacter*, and *Pseudarthrobacter*, were keystone genera in the endosphere of healthy tobacco plants and may have been involved in maintaining rhizosphere health.

The high node degree (24) and higher abundance of *Ralstonia* in the endosphere of diseased tobacco plants indicate that *Ralstonia* is a highly connected and important taxon within the network. Correlations between *Ralstonia* and other microbial members were calculated to identify critical species (Fig. 5C and S5C). More connections were found in diseased roots (26 bacterial and 4 fungal genera) than in healthy roots (19 bacterial and 2 fungal genera). Specifically, all 30 microbial genera were negatively related to *Ralstonia* in the diseased endosphere; nine microbial genera related to *Ralstonia* tended to co-exclude (negative correlations), and 12 genera co-occurred (positive correlations; Fig. S5C) in the healthy endosphere. Moreover, four genera, including *Pseudomonas*, *Sphingopyxis*, *Bosea*, and *Sphingobacterium*, were negatively correlated with *Ralstonia* in diseased and healthy roots. Furthermore, some bacterial genera, including *Sphingomonas*, *Ensifer*, *Allorhizobium*-*Neorhizobium-Pararhizobium*-*Rhizobium*, *Streptomyces*, and *Flavobacterium*, and fungal taxa, including *Alternaria*, *Coprinopsis*, and *Rhizodermea*, were negatively correlated with *Ralstonia* in diseased root samples, indicating that these microbial groups may participate in suppressing the growth of pathogens (Fig. 5C).

### Specialized function profiles of microbiomes between diseased and healthy tobacco plants

**Fig. 6:**
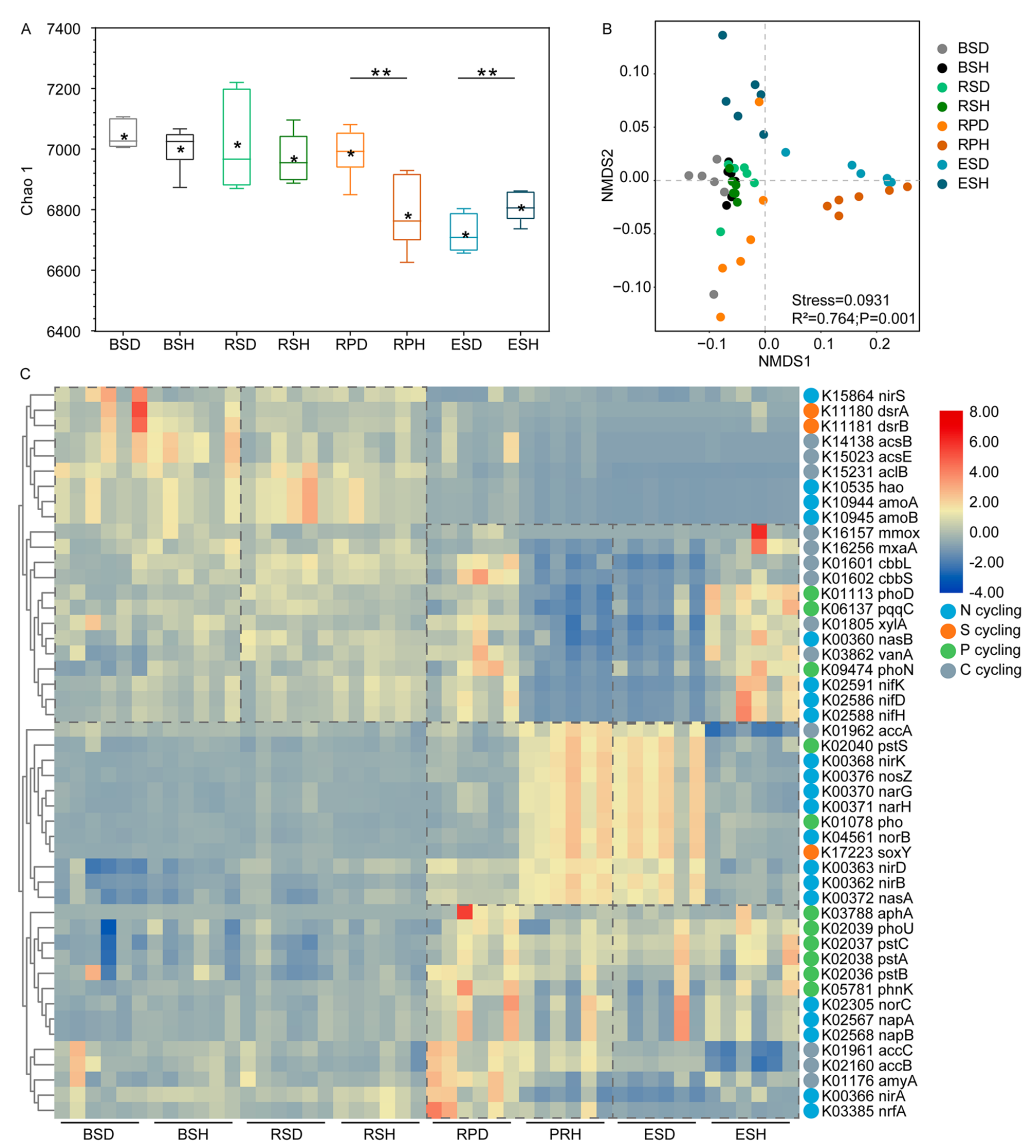
PICRUSt predicted functions of bacteria at KO level. **(A)** Chao 1 index showed the functional diversities of KO profiles in each compartment of healthy and diseased tobacco plants. Asterisks (**, *P* < 0.01) indicate significant differences. **(B)** Ordination of functional genes using NMDS on KO level. **(C)** Heatmap exhibited the relative abundance of functional genes involved in carbon, nitrogen, and phosphorus cycling in each compartment. BS: bulk soils; RS: rhizosphere; RP: rhizoplane; ES: endosphere; D: diseased tobacco plants; H: healthy tobacco plants

The potential ecological roles of microbiomes under disease conditions were predicted using PICRUSt2. Overall, 7,620 KEGG orthologs were predicted in the healthy and diseased bacterial communities. Consistent with the alpha diversity of the bacterial community, bacterial communities of the healthy endosphere had higher functional diversity than that of the diseased endosphere (Fig. 6A, *P* < 0.05, t-test). However, the rhizoplane microbiome in diseased tobacco plants possessed higher functional diversity than that in the healthy groups (Fig. 6A, *P* < 0.05, t-test). Additionally, the functional compositions of the bacterial community differed significantly across compartments (Fig. 6B and Table S4, PERMANOVA, *P* < 0.05), and disease significantly influenced bacterial functions in the three root-associated niches excluding the bulk soils (Table S4; PERMANOVA, *P* < 0.05). To identify the specialized functions of bacterial communities of diseased and healthy tobacco plants, the genes involved in carbon, nitrogen, sulfur, and phosphate cycling were analyzed. Significantly different patterns were found among the four compartments of diseased and healthy groups (Fig. 6C). Specifically, the genes related to sulfur reduction (*dsrA* and *dsrB*) and nitrification (e.g., *amoA*, *amoB*, and *hao*) were higher in the microbiomes of bulk and rhizosphere soils, and genes involved in phosphate transport (e.g., *pstA*, *pstB*, and *pstC*), phosphatase (e.g., *aphA* and *pho*), nitrogen reduction (e.g., *napA* and *napB*) and denitrification (e.g., *nirA*, *aphA*, and *pho*) were more abundant in the microbiomes of rhizoplane and endosphere (Fig. 6C). Moreover, based on the PERMANOVA results, the disease had a larger influence on the microbiomes of endosphere and rhizoplane than on those of the bulk and rhizosphere soils (Fig. 6C and Table S4). Additionally, compared with the healthy endosphere, functional genes related to nitrogen fixation (e.g., *nifD*, *nifH*, and *nifK*), methanol metabolism (*mmox* and *mxaA*), carbon fixation (*cbbL* and *cbbS*), and carbon degradation (*xylA* and *vanA*) were deleted in diseased endosphere and genes involved in nitrogen reduction (e.g., *nasA*, and *nirB*) and denitrification (e.g., *nosZ*, and *nirK*) were enriched in the diseased endosphere (Fig. 6C).

**Fig. 7:**
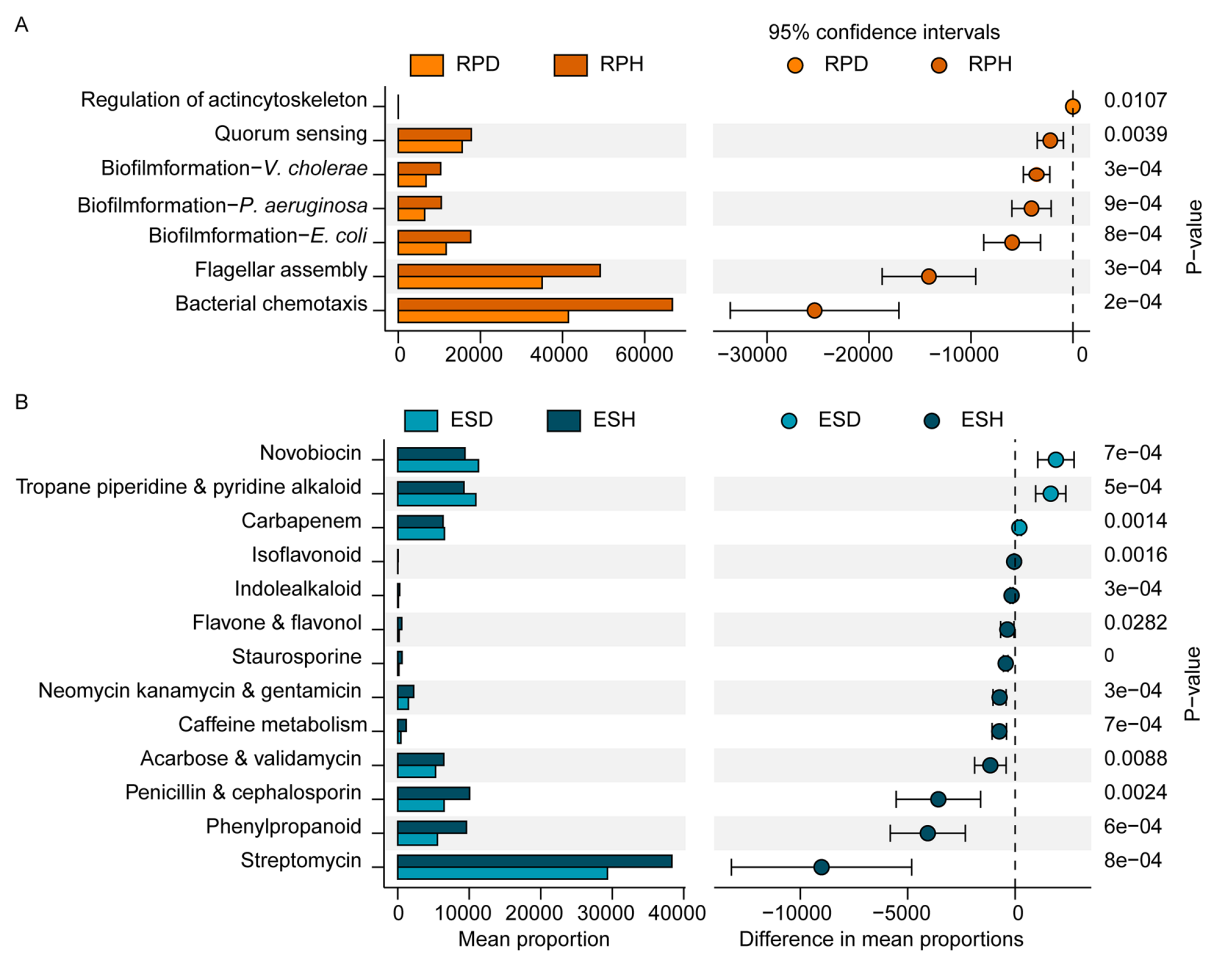
Difference in the relative abundance of bacterial functions involved in cellular processes and biosynthesis of other secondary metabolites between healthy and diseased rhizoplane **(A)** or endosphere **(B)** at KEGG level 3. RP: rhizoplane; ES: endosphere; D: diseased tobacco plants; H: healthy tobacco plants

To provide deeper insights into the influence of diseases on bacterial function, variances in the abundance of KEGG pathways between diseased and healthy tobacco plants were calculated. Seventeen functional modules (including amino acid metabolism, xenobiotic biodegradation and metabolism, and cell motility) were enriched in the healthy rhizoplane, five KEGG modules (such as glycan biosynthesis and metabolism, and biosynthesis of other secondary metabolites) were enriched in the healthy endosphere (Fig. S6). Moreover, we compared the functional genes involved in cellular processes and biosynthesis of other secondary metabolites that may participate in maintaining healthy tobacco plants. The pathways involved in biofilm formation, flagellar assembly, chemotaxis, and quorum sensing were enriched in the healthy rhizoplane, and some pathways related to antibiotic synthesis (e.g., streptomycin, penicillin, neomycin, and kanamycin) were enriched in the healthy endosphere (Fig. 7).

Furthermore, the functions of the fungal community were predicted using the ITS data, and 72 pathways were identified. Significant variations in the functional profiles of the fungal community were found between diseased and healthy tobacco plants, especially for rhizosphere fungi, in which 29 pathways were enriched or deleted in healthy tobacco plants (Fig. S7). Moreover, eight and four pathways were enriched in the healthy rhizoplane and endosphere, respectively, including methyl ketone biosynthesis, formaldehyde assimilation, and palmitate biosynthesis, amongst others (Fig. S7), which may boost plant defenses against pathogens.

## Discussion

An imbalance in rhizosphere microbial community structure is the main cause of bacterial wilt in crops. Identifying the changes in the community compositions and assembly processes of microbiomes under disease invasion is crucial for better understanding of how microorganisms maintain plant health and how plant microorganism interactions co-evolve under different conditions. Our results showed that microbial community assembly was affected by pathogen infections and ecological niches, and the endosphere and rhizoplane microbiomes were more susceptible to bacterial wilt. Drift was the major contributor to the microbial community assembly of diseased tobacco plants in the endosphere and rhizoplane compartments. Additionally, we identified the keystone species that may participate in defending against disease and revealed different potential defense mechanisms by which the endosphere and rhizoplane microbiome help plants maintain their health under disease stress.

### Distinct microbial community composition and assembly shaped by plant disease

Exploring microbial abundance and community composition is important to understand the microbial assembly process in different compartments under disease stress. The absolute abundances of microbial abundance in endosphere and rhizoplane were higher than that in bulk and rhizosphere soils, which may be caused by the higher contents and diversity of metabolites secreted by plants closer to the roots [1]. In addition, we found the microbial population in roots were more abundant than that in the rhizosphere soil, which was consistent with a previous report [15]. Compared to soil, the volume of roots per unit mass is larger, which could provide greater living space for microorganisms. However, it is also possible that there is a certain proportion of plant DNA contamination in the quantification of endophytic microorganisms with the limitation of primer specificity, due to the high sequence homology between plant DNA and bacterial and fungal DNA [41, 42]. Consistent with the field phenotype, the absolute and relative abundances of pathogenic *Ralstonia* in root and root-associated soils of diseased tobacco plants were higher than those in healthy tobacco plants (Fig. 1 C), confirming the occurrence of bacterial wilt. Moreover, a higher absolute and relative abundance of pathogenic *Fusarium* were also found in diseased samples, consistent with a previous study [15]. High abundances of pathogenic *Ralstonia* and *Fusarium* indicated a high possibility of mixed occurrence of bacterial wilt and *Fusarium* root rot of tobacco [43]. Consistent with previous reports [11, 15], the disease invasion strongly influenced microbial community diversity, with a lower alpha diversity in diseased samples, especially in the compartments close to or in the roots (Fig. 2A-D). Furthermore, high bacterial diversity is related to enhanced resistance to pathogen invasion and plant infestation [7]. The diversity of microbial communities was lower in the rhizoplane and endosphere niches than in the bulk and rhizosphere soils, which may be beneficial for pathogen invasion [44]. Additionally, microbial diversities and community compositions were more significantly influenced by the niches than by the plant’s health conditions (Fig. 2A-D; Table 1). Besides different health conditions [11], some studies focused on other factors, such as host [3, 32] and soil types [17], revealing an obvious gradient from the bulk soils to the endosphere niches in alpha diversity and significant variances across different niches in microbial structure (beta diversity). This indicates that plant compartments are vital to plant-related microbial community assembly [19].

Unlike aquatic microorganisms, the dispersal ability of soil microorganisms may be lower, which may strengthen the effect of stochasticity on community assembly. In the present study, stochastic processes (dispersal limitation and drift) dominated the assembly processes in all niches (Fig. 2G and H). This contradicts previous reports that the microbial community in the rhizosphere is mainly affected by deterministic processes in rice and maize [16, 18]. Microbial assembly processes are influenced by many factors, including environmental factors, geographic scale, and plant growth stages [18, 45]. In the present study, stronger stochasticity indicated small environmental gradients across these root-associated niches or weak impacts of the environmental change [46]. However, deterministic processes contributed more to endosphere bacterial community assembly than stochastic processes, consistent with previous studies showing that host selection stress was higher in plants than in soil [18, 47]. The soil is a crucial “seed bank” for plant microbiomes, containing various microorganisms [48]. In our study, soils were the major sources of microbial communities in the four niches (Fig. S1). However, microbial sources of the rhizoplane and endosphere communities were influenced by the diseases. While the major sources of the endosphere bacterial community were the rhizoplane (39.3%), rhizosphere (38.8%), and bulk soils (17.0%) in healthy tobacco plants, the contribution of the bulk (1.5%) and rhizosphere soils (13.3%) decreased in infected tobacco plants. These results are inconsistent with a recent study that pathogen invasion increased the contribution of the bulk and rhizosphere soils to the endosphere bacterial community [11]. Additionally, the major source of rhizoplane microbiomes was the endosphere in the diseased samples. The communication of bacterial communities is closer within the endosphere and rhizoplane in diseased tobacco plants, possibly because of pathogen invasion, which leads to the outflow of microbiomes within the roots.

Furthermore, microbial interactions were affected by disease invasion. In particular, the co-occurrence networks of healthy samples were more complex, especially in the endosphere and rhizoplane, with higher numbers of nodes, edges, and average degrees compared with the networks of diseased samples (Fig. 5). According to previous reports, complex networks are more robust than simple networks in facing external stresses such as environmental changes and pathogen invasion [49, 50]. Our results are consistent with previous researches [11, 13, 15], indicating that highly connected networks might help plants suppress disease invasion. Additionally, the positive interactions between microbes were stronger in diseased tobacco plants than in healthy plants, especially in the endosphere and rhizoplane (Fig. 5). Positive correlations between different microbes at the same nutritional level indicate a facilitation relationship, which is mutually beneficial to the interacting partners [51]. It was suggested that the positive or negative correlations between microbial taxa and pathogens were considered as “pathogen facilitators” or “pathogen antagonists,” respectively [52, 53]. More connections between the microbial genera and the pathogen *Ralstonia* were found in the diseased endosphere, and nearly all correlations were negative in the diseased endosphere (Fig. 5 and S5). Overall, these observations of the enhancement of facilitation among other microbial taxa in diseased plants might indicate a combination of individual taxa to defend against disease invasion. More experiments in vitro on the impact of putative keystone taxon will further explore and verify their function in defending against disease.

### Varied defense mechanisms of keystone taxa against diseases in different niches

It has been suggested that the active root-related microbiome could boost plants’ ability to cope with external biological and abiotic pressures [4, 54]. Plants seek to recruit a beneficial consortium of microbiomes using chemical substrates during pathogen invasion [9, 10]. For example, tomato root exudation of sucrose enhanced the colonization of *B. subtilis*, which significantly improved biocontrol efficiency against *Fusarium* wilt and gray mold [9]. In our study, a variety of microbes were enriched in diseased tobacco plants, and the members were different across the niches (Fig. 4). This is consistent with previous reports where it was shown that plants might be able to filter certain microbes or that different microorganisms tend to be enriched in favorable colonization niches [3]. For example, the genera *Rhodococcus*, *Flavobacterium*, and *Sphingobacterium* in rhizoplane and *Bacillus* and *Pseudomonas* in the endosphere were enriched in diseased tobacco plants, most of which antagonized *Fusarium* wilt or bacterial wilt. While *Bacillus* and *Pseudomonas* were found to directly kill the pathogens by secreting some active substances [55, 56], and some bacteria, as a role of helper bacteria, indirectly increased control efficiency of disease. Some species of *Flavobacterium* genus could increase biofilm formation of biocontrol *Bacillus velezensis* and elevate the transcription of plant defense gene to improve the control efficiency of tomato bacterial wilt [57]. In addition, *Sphingobacterium tabacisoli* showed the first line of defense against *Fusarium* wilt by accumulating defense enzymes such as peroxidase, and polyphenol oxidase and triggering systemic resistance of banana [58]. Hence, when faced with pathogen invasion, the recruitment of beneficial microbial strains by plants may reduce the harm the disease causes [8]. This is confirmed by the correlations between microbes and pathogenic *Ralstonia* in the endosphere, with more microbes negatively correlated with *Ralstonia* in the diseased tobacco plants (Fig. 5 and S5). Besides four genera (*Pseudomonas*, *Sphingobacterium*, *Sphingopyxis*, and *Bosea*) in healthy and diseased endospheres, 17 unique genera such as *Stenotrophomonas, Sphingomonas*, *Ensifer*, *Streptomyces*, *Chryseobacterium*, *Klebsiella* and *Flavobacterium*, were negatively correlated with *Ralstonia* in the diseased endosphere, which might participate in defending against *Ralstonia* invasion in multiple ways. For example, *Streptomyces, Stenotrophomonas*, *Chryseobacterium*, and *Klebsiella* were reported to effectively inhibit the growth of pathogenic *Ralstonia* by using plate inhibition assays and pot experiments [11, 59–61]. Additionally, *Sphingomonas* sp. Cra20 offers plants considerable protection against pathogenic *Ralstonia* by interfering the virulence-related genes of *R. solanacearum* and reshaping the transcriptomes of the susceptible tomatoes [62]. Moreover, the genes involved in nitrogen reduction and denitrification (*nasA*, *nirB*, *nosZ*, and *nirK*) were enriched in diseased tobacco plants, which was consistent to a previous study [63]. Nitrogen reduction and denitrification convert available nitrogen into unusable nitrogen for plants, causing a loss of nitrogen sources [64]. Hence, changes in microbial structures lead to an imbalance in community function, which may cause a chain reaction and further reduce the ability of plants to autonomously defend against diseases.

Indicator taxa are crucial for the robustness of community structures and play pivotal roles in community function [65]. Indicator genera were identified in healthy and diseased tobacco plants using linear discriminant analysis effect size and network analyses (Fig. 5C). The bacterial ASVs belonging to Burkholderiales_SC-I-84, *Burkholderia*-*Caballeronia*-*Paraburkholderia*, Gemmatimonadaceae, *Chryseobacterium*, *Haliangium*, *Allorhizobium*-*Neorhizobium*-*Pararhizobium*-*Rhizobium* were abundant (0.13–0.79%) in healthy endosphere and rhizoplane (Fig. 4D and S4), which might be good predictors of plant’s health. In previous studies, the above six bacterial taxa were plant growth-promoting bacteria and were regarded as the node hubs or indicators for other plant species [66–68]. In our study, the keystone species of the rhizoplanes and endospheres in healthy tobacco plants included Gemmatimonadaceae, *Haliangium*, and *Allorhizobium*-*Neorhizobium*-*Pararhizobium*-*Rhizobium*, indicating the crucial roles of these microorganisms in the balance of community function and pathogen defense.

Moreover, the analysis of community functions indicated that microbial functional genes involved in the cellular community and cell motility were enriched in the healthy rhizoplane, and the genes related to the biosynthesis of other secondary metabolites were more abundant in the healthy endosphere than in diseased endosphere (Fig. S6), indicating varied pathogen defense strategies of the microbiome in different niches. Previous studies suggested that biofilm formation on plant roots can improve the colonization of biocontrol agents and the inhibition efficiency of disease [9, 69]. To defend against pathogen invasion, microbes in healthy rhizoplanes might seize ecological niches on the surface of the root by forming biofilms, evidenced by the higher abundance of genes related to flagellar assembly and biofilm formation in healthy rhizoplanes than in diseased rhizoplanes (Fig. 7). Additionally, the microbial functions of chemotaxis and quorum sensing were enriched in the healthy rhizoplanes (Fig. 7A), which may promote biofilm formation [70]. Unlike microorganisms on the surface of roots, microbes in the endosphere adopt a more direct approach to defend against pathogen invasion. The abundance of pathways related to the biosynthesis of other secondary metabolites, such as streptomycin, penicillin, and validamycin, was significantly higher in the healthy endosphere than in the diseased endosphere (Fig. 7B). In previous studies, streptomycin, penicillin, and validamycin killed *R. solanacearum* and effectively prevented and controlled bacterial wilt [71, 72]. Furthermore, the microbial functions of nitrogen and carbon fixation were enriched in healthy tobacco plants, which might promote plant growth and indirectly enhance the disease-resistance ability of plants. Collectively, our findings could enhance the understanding of the microbial assembly mechanisms and the varied defense strategies of plants under pathogen invasion. Compared to a healthy microbial community, it is more possible to identify the indicator biocontrol agents in diseased groups based on the network analysis. However, the relationships between microbial taxa in cooperation or competition have not been fully elucidated and need to be further verified to design synthetic microbial communities for sustainable green agriculture under pathogen invasion [73].

## Conclusions

Overall, our work investigated the microbial structure and predicted the functional profiles of root-related microbiomes affected by bacterial wilt, revealing distinct microbial composition and potential defense strategies in different niches. Microbial diversity was significantly reduced during pathogen invasion, and ecological niche and pathogen invasion influenced the microbial assembly process. Additionally, in healthy tobacco plants, some microbes in the rhizoplane formed the first line of defense by forming biofilms to occupy ecological niches, the endosphere microbiomes suppressed the plants by producing antibiotics to kill the pathogens, and some nitrogen and carbon fixation microbes fixed the nitrogen and carbon in the endosphere to enhance plant growth. Diseased tobacco plants resisted pathogen invasion by recruiting some “pathogen antagonists,” including *Flavobacterium*, *Stenotrophomonas*, and *Pseudomonas*. Our findings highlight the assembly and functions of the microbial community in the soil-root continuum in response to pathogen stresses and lay the foundation for the possible use of key microbiomes to suppress plant diseases.

### Electronic supplementary material

Below is the link to the electronic supplementary material.

Supplementary Material 1

## Data Availability

The raw sequence data in this study are deposited in the NCBI Sequence Read Archive (SRA) database under the accession numbers PRJNA987547 and PRJNA987752.
