# Supplementary material for: Bacterial wilt affects the structure and assembly of microbial communities along the soil-root continuum
Source: Environ Microbiome. 2024 Jan 16;19:6. doi: 10.1186/s40793-024-00548-7 (PMC10792853; doi:10.1186/s40793-024-00548-7)
Supplement: Supplementary file 1 — Supplementary Material 1 [file 40793_2024_548_MOESM1_ESM.docx]

Supplementary material for

**Bacterial wilt affects the structure and assembly of microbial communities along the soil-root continuum**

**Table S1** The richness (Chao I and ACE) indices, diversity (Shannon and Simpson) indices and Good’s coverage across all samples

| Sample | Bacteria | | | | | Fungi | | | | |
| --- | --- | --- | --- | --- | --- | --- | --- | --- | --- | --- |
|  | Shannon | Simpson | Chao1 | ACE | Good’s coverage | Shannon | Simpson | Chao1 | ACE | Good’s coverage |
| BSD1 | 5.24 | 0.99 | 822 | 824 | 0.9995 | 2.97 | 0.90 | 238 | 239 | 0.9999 |
| BSD2 | 4.27 | 0.90 | 796 | 797 | 0.9993 | 2.68 | 0.87 | 222 | 224 | 0.9999 |
| BSD3 | 4.57 | 0.94 | 825 | 816 | 0.9992 | 3.45 | 0.94 | 283 | 282 | 0.9998 |
| BSD4 | 5.28 | 0.99 | 936 | 936 | 0.9990 | 3.46 | 0.94 | 279 | 278 | 0.9998 |
| BSD5 | 5.13 | 0.98 | 899 | 899 | 0.9991 | 3.16 | 0.92 | 290 | 290 | 0.9998 |
| BSD6 | 5.62 | 0.99 | 965 | 966 | 0.9993 | 3.21 | 0.92 | 249 | 250 | 0.9999 |
| BSH1 | 5.44 | 0.99 | 817 | 818 | 0.9997 | 3.39 | 0.93 | 276 | 275 | 0.9999 |
| BSH2 | 5.24 | 0.99 | 788 | 793 | 0.9994 | 3.47 | 0.93 | 267 | 267 | 0.9999 |
| BSH3 | 5.25 | 0.99 | 784 | 785 | 0.9996 | 3.61 | 0.94 | 290 | 288 | 0.9999 |
| BSH4 | 5.00 | 0.98 | 749 | 751 | 0.9996 | 3.04 | 0.90 | 274 | 275 | 0.9998 |
| BSH5 | 4.97 | 0.98 | 806 | 806 | 0.9993 | 3.22 | 0.92 | 258 | 259 | 0.9999 |
| BSH6 | 5.46 | 0.99 | 871 | 873 | 0.9994 | 3.35 | 0.93 | 291 | 291 | 0.9999 |
| RSD1 | 4.64 | 0.96 | 779 | 775 | 0.9991 | 2.25 | 0.77 | 241 | 241 | 0.9998 |
| RSD2 | 4.98 | 0.98 | 862 | 859 | 0.9991 | 2.18 | 0.74 | 237 | 238 | 0.9998 |
| RSD3 | 5.10 | 0.98 | 762 | 764 | 0.9995 | 2.70 | 0.87 | 228 | 230 | 0.9999 |
| RSD4 | 5.15 | 0.99 | 818 | 813 | 0.9990 | 2.21 | 0.75 | 218 | 221 | 0.9998 |
| RSD5 | 5.03 | 0.98 | 754 | 759 | 0.9994 | 2.51 | 0.82 | 210 | 209 | 0.9998 |
| RSD6 | 5.09 | 0.98 | 858 | 859 | 0.9991 | 1.52 | 0.52 | 181 | 184 | 0.9997 |
| RSH1 | 5.18 | 0.99 | 775 | 777 | 0.9996 | 3.08 | 0.89 | 273 | 271 | 0.9999 |
| RSH2 | 5.20 | 0.99 | 824 | 827 | 0.9994 | 3.64 | 0.94 | 307 | 301 | 0.9998 |
| RSH3 | 4.97 | 0.98 | 782 | 781 | 0.9994 | 3.71 | 0.95 | 326 | 327 | 0.9999 |
| RSH4 | 5.02 | 0.98 | 784 | 784 | 0.9995 | 3.56 | 0.95 | 280 | 279 | 0.9999 |
| RSH5 | 5.00 | 0.98 | 798 | 800 | 0.9994 | 3.53 | 0.94 | 290 | 289 | 0.9999 |
| RSH6 | 4.92 | 0.98 | 754 | 758 | 0.9995 | 3.54 | 0.95 | 321 | 322 | 0.9999 |
| RPD1 | 2.84 | 0.77 | 624 | 624 | 0.9989 | 1.74 | 0.56 | 240 | 242 | 0.9998 |
| RPD2 | 2.04 | 0.66 | 464 | 470 | 0.9989 | 1.77 | 0.62 | 205 | 201 | 0.9997 |
| RPD3 | 2.12 | 0.59 | 528 | 528 | 0.9991 | 1.82 | 0.66 | 211 | 215 | 0.9997 |
| RPD4 | 0.79 | 0.20 | 540 | 532 | 0.9983 | 2.50 | 0.81 | 249 | 253 | 0.9997 |
| RPD5 | 2.56 | 0.71 | 668 | 679 | 0.9987 | 2.31 | 0.75 | 260 | 262 | 0.9997 |
| RPD6 | 1.40 | 0.38 | 442 | 449 | 0.9989 | 2.13 | 0.70 | 237 | 234 | 0.9998 |
| RPH1 | 3.65 | 0.86 | 659 | 659 | 0.9992 | 2.87 | 0.86 | 259 | 260 | 0.9998 |
| RPH2 | 4.22 | 0.92 | 717 | 720 | 0.9992 | 2.98 | 0.85 | 303 | 303 | 0.9998 |
| RPH3 | 4.48 | 0.97 | 671 | 672 | 0.9993 | 2.92 | 0.90 | 235 | 235 | 0.9999 |
| RPH4 | 4.35 | 0.96 | 627 | 626 | 0.9992 | 2.89 | 0.86 | 242 | 244 | 0.9999 |
| RPH5 | 4.20 | 0.92 | 760 | 747 | 0.9987 | 2.91 | 0.87 | 246 | 248 | 0.9999 |
| RPH6 | 4.66 | 0.97 | 713 | 717 | 0.9994 | 2.15 | 0.73 | 168 | 168 | 0.9999 |
| ESD1 | 2.27 | 0.65 | 394 | 395 | 0.9992 | 2.41 | 0.82 | 133 | 135 | 0.9999 |
| ESD2 | 1.35 | 0.39 | 400 | 403 | 0.9990 | 1.85 | 0.74 | 121 | 124 | 0.9998 |
| ESD3 | 2.23 | 0.63 | 400 | 405 | 0.9992 | 2.14 | 0.82 | 141 | 140 | 0.9998 |
| ESD4 | 1.28 | 0.36 | 367 | 360 | 0.9991 | 2.13 | 0.83 | 130 | 126 | 0.9997 |
| ESD5 | 3.49 | 0.93 | 432 | 429 | 0.9993 | 2.19 | 0.80 | 130 | 131 | 0.9998 |
| ESD6 | 1.20 | 0.34 | 331 | 324 | 0.9992 | 2.19 | 0.81 | 135 | 137 | 0.9998 |
| ESH1 | 3.27 | 0.88 | 470 | 464 | 0.9990 | 2.53 | 0.87 | 132 | 131 | 0.9999 |
| ESH2 | 3.58 | 0.93 | 484 | 485 | 0.9990 | 2.23 | 0.83 | 113 | 114 | 0.9999 |
| ESH3 | 3.86 | 0.96 | 396 | 391 | 0.9994 | 2.05 | 0.81 | 101 | 103 | 0.9999 |
| ESH4 | 4.22 | 0.97 | 496 | 492 | 0.9991 | 3.11 | 0.91 | 248 | 251 | 0.9997 |
| ESH5 | 3.89 | 0.94 | 459 | 463 | 0.9995 | 2.90 | 0.90 | 176 | 176 | 0.9999 |
| ESH6 | 3.71 | 0.93 | 506 | 510 | 0.9992 | 2.24 | 0.84 | 129 | 127 | 0.9998 |

BS, bulk soils; RS, rhizosphere; RP, rhizoplane; ES, endosphere; D, diseased tobacco plants; H, healthy tobacco plants.

**Table S2** Topological features of the co-occurrence bacterial networks of diseased and healthy tobacco plants

|  | Topological parameter | Bulk soils | Rhizosphere | Rhizoplane | Endosphere |
| --- | --- | --- | --- | --- | --- |
| Diseased | Node | 148 | 145 | 51 | 50 |
|  | Edge | 1887 | 2064 | 385 | 360 |
|  | Positive correlation proportion | 0.705 | 0.582 | 0.930 | 0.914 |
|  | Average degree | 25.500 | 28.469 | 15.098 | 14.400 |
|  | Average clustering coefficient | 0.567 | 0.594 | 0.593 | 0.583 |
|  | Average path distance | 2.237 | 2.139 | 2.036 | 2.178 |
| Healthy | Node | 158 | 143 | 115 | 96 |
|  | Edge | 2428 | 1428 | 1406 | 886 |
|  | Positive correlation proportion | 0.558 | 0.542 | 0.615 | 0.526 |
|  | Average degree | 30.734 | 19.972 | 24.452 | 18.458 |
|  | Average clustering coefficient | 0.597 | 0.494 | 0.603 | 0.569 |
|  | Average path distance | 2.128 | 1.985 | 2.122 | 2.186 |

**Table S3** Keystone taxa identified in the co-occurrence network of each group.

| ID | Degree | Betweenness centrality | Group | Taxonomy |
| --- | --- | --- | --- | --- |
| BSD-1 | 42 | 46.18122 | BSD | d__Bacteria;p__Acidobacteriota;c__Blastocatellia;o__11-24;f__11-24;g__11-24 |
| BSD-2 | 46 | 59.37061 | BSD | d__Bacteria;p__Acidobacteriota;c__Subgroup_5;o__Subgroup_5;f__Subgroup_5;g__Subgroup_5 |
| BSD-3 | 46 | 59.37061 | BSD | d__Bacteria;p__Acidobacteriota;c__Thermoanaerobaculia;o__Thermoanaerobaculales;f__Thermoanaerobaculaceae;  g__Subgroup_10 |
| BSD-4 | 46 | 59.37061 | BSD | d__Bacteria;p__Actinobacteriota;c__Actinobacteria;o__Propionibacteriales;f__Nocardioidaceae;g__Kribbella |
| BSD-5 | 46 | 59.37061 | BSD | d__Bacteria;p__Actinobacteriota;c__Actinobacteria;o__Pseudonocardiales;f__Pseudonocardiaceae;  g__Lechevalieria |
| BSD-6 | 42 | 46.18122 | BSD | d__Bacteria;p__Proteobacteria;c__Alphaproteobacteria;o__Rhizobiales;f__KF-JG30-B3;g__KF-JG30-B3 |
| BSD-7 | 46 | 59.37061 | BSD | d__Bacteria;p__Proteobacteria;c__Gammaproteobacteria;o__Burkholderiales;f__Rhodocyclaceae;g__Azoarcus |
| BSD-8 | 42 | 46.18122 | BSD | d__Bacteria;p__Proteobacteria;c__Gammaproteobacteria;o__Burkholderiales;f__TRA3-20;g__TRA3-20 |
| BSD-9 | 46 | 59.37061 | BSD | d__Bacteria;p__Proteobacteria;c__Gammaproteobacteria;o__CCD24;f__CCD24;g__CCD24 |
| BSD-10 | 42 | 46.18122 | BSD | d__Bacteria;p__Verrucomicrobiota;c__Verrucomicrobiae;o__Pedosphaerales;f__Pedosphaeraceae;  g__ADurb.Bin063-1 |
| BSD-11 | 46 | 59.37061 | BSD | d__Bacteria;p__Verrucomicrobiota;c__Verrucomicrobiae;o__Pedosphaerales;f__Pedosphaeraceae;  g__Pedosphaeraceae |
| BSH-1 | 49 | 91.28374 | BSH | d__Bacteria;p__Actinobacteriota;c__Actinobacteria;o__Micrococcales;f__Micrococcaceae;g__Paenarthrobacter |
| BSH-2 | 53 | 119.8812 | BSH | d__Bacteria;p__Bacteroidota;c__Bacteroidia;o__Sphingobacteriales;f__env.OPS_17;g__env.OPS_17 |
| BSH-3 | 48 | 88.05326 | BSH | d__Bacteria;p__Proteobacteria;c__Alphaproteobacteria;o__Sphingomonadales;f__Sphingomonadaceae;  g__Sphingobium |
| BSH-4 | 49 | 92.47791 | BSH | d__Bacteria;p__Proteobacteria;c__Gammaproteobacteria;o__Burkholderiales;f__Comamonadaceae__ |
| BSH-5 | 48 | 88.05326 | BSH | d__Bacteria;p__Proteobacteria;c__Gammaproteobacteria;o__Burkholderiales;f__Oxalobacteraceae;  g__Noviherbaspirillum |
| BSH-6 | 49 | 91.28374 | BSH | d__Bacteria;p__Proteobacteria;c__Gammaproteobacteria;o__Burkholderiales;f__Rhodocyclaceae;g__Azoarcus |
| BSH-7 | 53 | 119.8812 | BSH | d__Bacteria;p__Proteobacteria;c__Gammaproteobacteria;o__Burkholderiales;f__SC-I-84;g__SC-I-84 |
| BSH-8 | 49 | 91.28374 | BSH | d__Bacteria;p__Proteobacteria;c__Gammaproteobacteria;o__Pseudomonadales;f__Pseudomonadaceae;  g__Pseudomonas |
| BSH-9 | 48 | 88.05326 | BSH | k__Fungi;p__Ascomycota;c__Eurotiomycetes;o__Eurotiales;f__Trichocomaceae;g__Talaromyces |
| BSH-10 | 48 | 88.05326 | BSH | k__Fungi;p__Ascomycota;c__Sordariomycetes;o__Hypocreales;f__Hypocreales_fam_Incertae_sedis;  g__Acremonium |
| BSH-11 | 50 | 109.0047 | BSH | k__Fungi;p__Ascomycota;c__Sordariomycetes;o__Sordariales;f__Chaetomiaceae;g__Chaetomium |
| RSD-1 | 53 | 69.58086 | RSD | d__Bacteria;p__Acidobacteriota;c__Acidobacteriae;o__Acidobacteriales____ |
| RSD-2 | 53 | 69.58086 | RSD | d__Bacteria;p__Chloroflexi;c__Dehalococcoidia;o__S085;f__S085;g__S085 |
| RSD-3 | 51 | 105.4505 | RSD | d__Bacteria;p__Proteobacteria;c__Alphaproteobacteria;o__Rhizobiales;f__Rhizobiaceae;  g__Allorhizobium-Neorhizobium-Pararhizobium-Rhizobium |
| RSD-4 | 55 | 98.38935 | RSD | d__Bacteria;p__Proteobacteria;c__Alphaproteobacteria;o__Rhizobiales;f__Rhizobiaceae;g__Ensifer |
| RSD-5 | 53 | 69.58086 | RSD | d__Bacteria;p__Proteobacteria;c__Alphaproteobacteria;o__Rhizobiales;f__Xanthobacteraceae;g__uncultured |
| RSD-6 | 51 | 105.4505 | RSD | k__Fungi;p__Ascomycota;c__Sordariomycetes;o__Glomerellales;f__Plectosphaerellaceae;g__Plectosphaerella |
| RSD-7 | 51 | 105.4505 | RSD | k__Fungi;p__Ascomycota;c__Sordariomycetes;o__Hypocreales;f__Hypocreaceae;g__Trichoderma |
| RSD-8 | 52 | 66.27731 | RSD | k__Fungi;p__Ascomycota;c__Sordariomycetes;o__Sordariales;f__Chaetomiaceae;g__Zopfiella |
| RSH-1 | 34 | 105.6511 | RSH | d__Bacteria;p__Actinobacteriota;c__Actinobacteria;o__Micrococcales;f__Intrasporangiaceae;  g__Intrasporangium |
| RSH-2 | 34 | 97.48904 | RSH | d__Bacteria;p__Actinobacteriota;c__Actinobacteria;o__Micrococcales;f__Intrasporangiaceae;g__Terrabacter |
| RSH-3 | 35 | 90.96888 | RSH | d__Bacteria;p__Actinobacteriota;c__Thermoleophilia;o__Solirubrobacterales;f__Solirubrobacteraceae;  g__Solirubrobacter |
| RSH-4 | 35 | 114.0275 | RSH | d__Bacteria;p__Bacteroidota;c__Bacteroidia;o__Chitinophagales;f__Chitinophagaceae__ |
| RSH-5 | 35 | 90.96888 | RSH | d__Bacteria;p__Bacteroidota;c__Bacteroidia;o__Chitinophagales;f__Chitinophagaceae;g__Flavisolibacter |
| RSH-6 | 35 | 121.4525 | RSH | d__Bacteria;p__Proteobacteria;c__Alphaproteobacteria;o__Rhizobiales;f__Xanthobacteraceae__ |
| RSH-7 | 34 | 105.6511 | RSH | k__Fungi;p__Ascomycota;c__Dothideomycetes;o__Pleosporales;f__Didymellaceae__ |
| RSH-8 | 35 | 90.96888 | RSH | k__Fungi;p__Ascomycota;c__Sordariomycetes;o__Chaetosphaeriales;f__Chaetosphaeriaceae__ |
| RSH-9 | 35 | 90.96888 | RSH | k__Fungi;p__Basidiomycota;c__Tremellomycetes;o__Tremellales;f__Trimorphomycetaceae;g__Saitozyma |
| RPD-1 | 29 | 37.5623 | RPD | d__Bacteria;p__Acidobacteriota;c__Acidobacteriae;o__Solibacterales;f__Solibacteraceae;  g__Candidatus_Solibacter |
| RPD-2 | 30 | 41.8127 | RPD | d__Bacteria;p__Actinobacteriota;c__Actinobacteria;o__Corynebacteriales;f__Mycobacteriaceae;  g__Mycobacterium |
| RPD-3 | 30 | 41.8127 | RPD | d__Bacteria;p__Gemmatimonadota;c__Gemmatimonadetes;o__Gemmatimonadales;f__Gemmatimonadaceae;  g__Gemmatimonas |
| RPD-4 | 29 | 37.5623 | RPD | d__Bacteria;p__Proteobacteria;c__Gammaproteobacteria;o__Burkholderiales;f__Burkholderiaceae;  g__Burkholderia-Caballeronia-Paraburkholderia |
| RPD-5 | 30 | 41.8127 | RPD | d__Bacteria;p__Proteobacteria;c__Gammaproteobacteria;o__Xanthomonadales;f__Xanthomonadaceae;  g__Stenotrophomonas |
| RPH-1 | 47 | 39.77868 | RPH | d__Bacteria;p__Actinobacteriota;c__Thermoleophilia;o__Gaiellales;f__uncultured;g__uncultured |
| RPH-2 | 55 | 68.09584 | RPH | d__Bacteria;p__Gemmatimonadota;c__Gemmatimonadetes;o__Gemmatimonadales;f__Gemmatimonadaceae;  g__Gemmatimonas |
| RPH-3 | 53 | 47.50127 | RPH | d__Bacteria;p__Gemmatimonadota;c__Gemmatimonadetes;o__Gemmatimonadales;f__Gemmatimonadaceae;  g__uncultured |
| RPH-4 | 53 | 47.50127 | RPH | d__Bacteria;p__Gemmatimonadota;c__S0134_terrestrial_group;o__S0134_terrestrial_group;  f__S0134_terrestrial_group;g__S0134_terrestrial_group |
| RPH-5 | 55 | 68.09584 | RPH | d__Bacteria;p__Myxococcota;c__Polyangia;o__Haliangiales;f__Haliangiaceae;g__Haliangium |
| RPH-6 | 47 | 77.26388 | RPH | k__Fungi;p__Ascomycota;c__Dothideomycetes;o__Pleosporales;f__Lentitheciaceae;g__Poaceascoma |
| RPH-7 | 47 | 77.26388 | RPH | k__Fungi;p__Ascomycota;c__Dothideomycetes;o__Pleosporales;f__Morosphaeriaceae;g__Acrocalymma |
| RPH-8 | 47 | 77.26388 | RPH | k__Fungi;p__Ascomycota;c__Sordariomycetes;o__Sordariales____ |
| RPH-9 | 53 | 47.50127 | RPH | k__Fungi;p__Ascomycota;c__Sordariomycetes;o__Sordariales;f__Chaetomiaceae__ |
| ESD-1 | 26 | 31.29559 | ESD | d__Bacteria;p__Proteobacteria;c__Alphaproteobacteria;o__Rhizobiales;f__Devosiaceae;g__Devosia |
| ESD-2 | 26 | 39.78386 | ESD | d__Bacteria;p__Proteobacteria;c__Alphaproteobacteria;o__Rhizobiales;f__Rhizobiaceae;g__Ensifer |
| ESD-3 | 27 | 32.4196 | ESD | d__Bacteria;p__Proteobacteria;c__Gammaproteobacteria;o__Enterobacterales;f__Enterobacteriaceae;  g__Klebsiella |
| ESD-4 | 26 | 39.78386 | ESD | d__Bacteria;p__Proteobacteria;c__Gammaproteobacteria;o__Pseudomonadales;f__Pseudomonadaceae;  g__Pseudomonas |
| ESH-1 | 43 | 60.84337 | ESH | d__Bacteria;p__Actinobacteriota;c__Actinobacteria;o__Micrococcales;f__Micrococcaceae;g__Pseudarthrobacter |
| ESH-2 | 43 | 74.92935 | ESH | d__Bacteria;p__Actinobacteriota;c__Thermoleophilia;o__Solirubrobacterales;f__Solirubrobacteraceae;  g__Conexibacter |
| ESH-3 | 43 | 60.84337 | ESH | d__Bacteria;p__Proteobacteria;c__Alphaproteobacteria;o__Rhizobiales;f__Rhizobiaceae;  g__Allorhizobium-Neorhizobium-Pararhizobium-Rhizobium |
| ESH-4 | 43 | 61.61567 | ESH | d__Bacteria;p__Proteobacteria;c__Gammaproteobacteria;o__Burkholderiales;f__Comamonadaceae__ |
| ESH-5 | 43 | 60.84337 | ESH | d__Bacteria;p__Proteobacteria;c__Gammaproteobacteria;o__Burkholderiales;f__Oxalobacteraceae__ |
| ESH-6 | 43 | 74.92935 | ESH | d__Bacteria;p__Proteobacteria;c__Gammaproteobacteria;o__Xanthomonadales;f__Xanthomonadaceae__ |

BS, bulk soils; RS, rhizosphere; RP, rhizoplane; ES, endosphere; D, diseased tobacco plants; H, healthy tobacco plants.

**Table S4** R^2^ and *P* values calculated by PERMANOVA test for the variance of function for bacterial communities among different samples

| Factor(s) | Results by factor | |  | Results by health condition/niche | | |
| --- | --- | --- | --- | --- | --- | --- |
|  | R^2^ | *P* value |  | Comparison | R^2^ | *P* value |
| Health condition | 0.010 | 0.667 |  | BSD vs BSH | 0.123 | 0.223 |
|  |  |  |  | RSD vs RSH | 0.237 | **0.015** |
|  |  |  |  | RPD vs RPH | 0.717 | **0.005** |
|  |  |  |  | ESD vs ESH | 0.698 | **0.004** |
| Niche | 0.362 | **0.001** |  | BSD vs RSD vs RPD vs ESD | 0.713 | **0.001** |
|  |  |  |  | BSH vs RSH vs RPH vs ESH | 0.819 | **0.001** |
| Health condition & niche | 0.764 | **0.001** |  |  |  |  |

*P* values in bold means significantly correlated (*P* < 0.05, n = 6). BS, bulk soils; RS, rhizosphere; RP, rhizoplane; ES, endosphere; D, diseased tobacco plants; H, healthy tobacco plants.


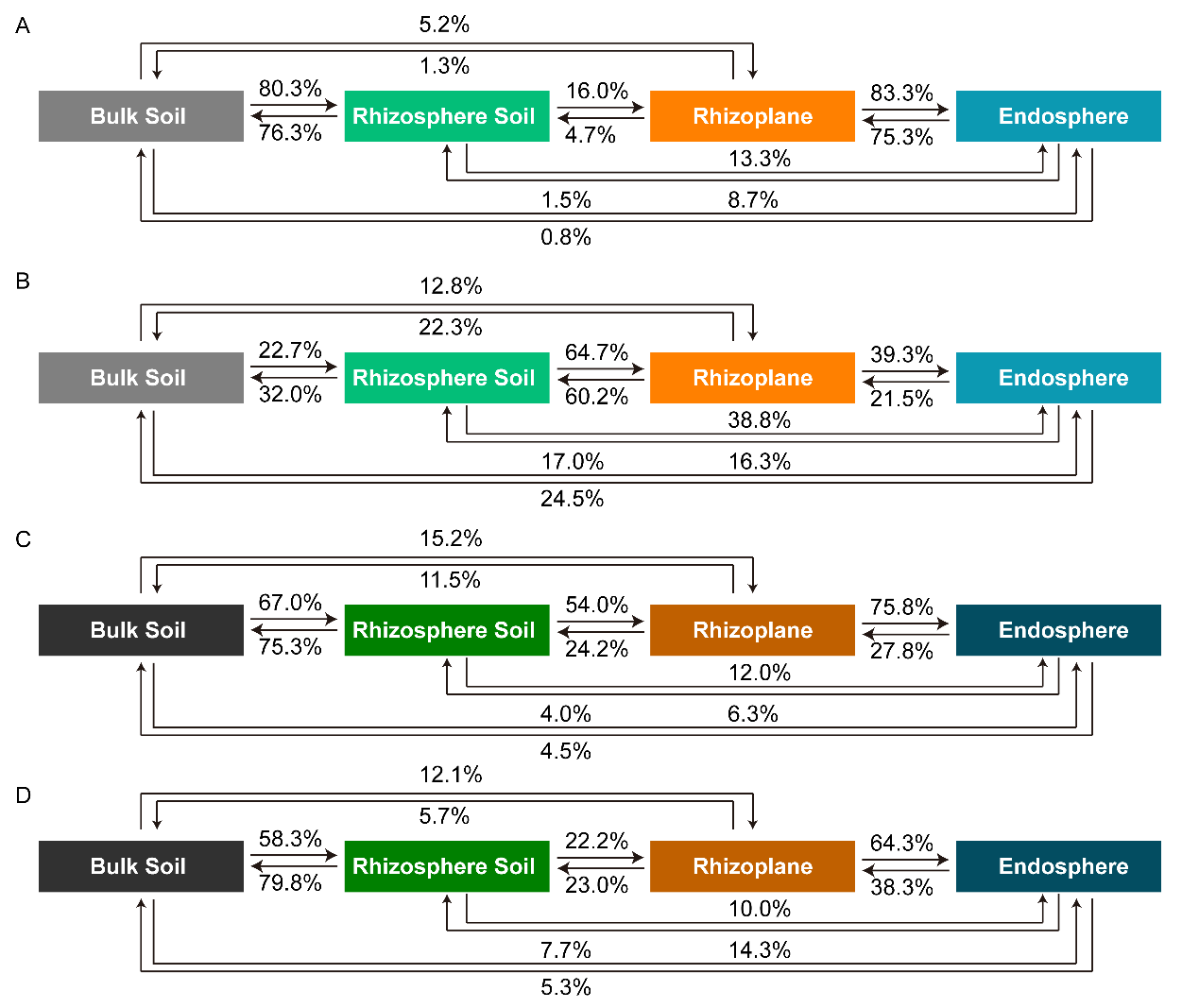


**Fig. S1** Source-tracking analysis showing the sources of the rhizosphere, rhizoplane, and endosphere bacterial species in diseased (A) and healthy tobacco plants (B). Source-tracking analysis showing the sources of the rhizosphere, rhizoplane, and endosphere fungal species in diseased (C) and healthy tobacco plants (D).


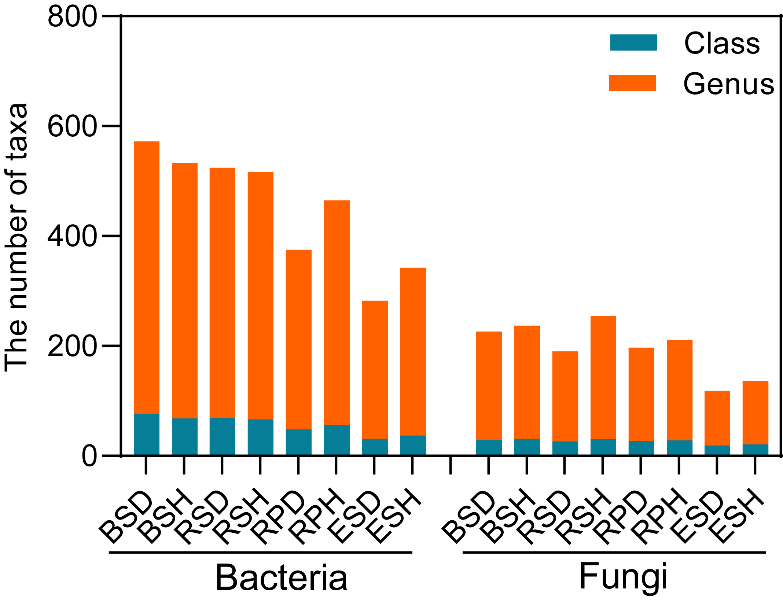


**Fig. S2** The number of bacterial and fungal taxa in different samples. BS, bulk soils; RS, rhizosphere; RP, rhizoplane; ES, endosphere; D, diseased tobacco plants; H, healthy tobacco plants.


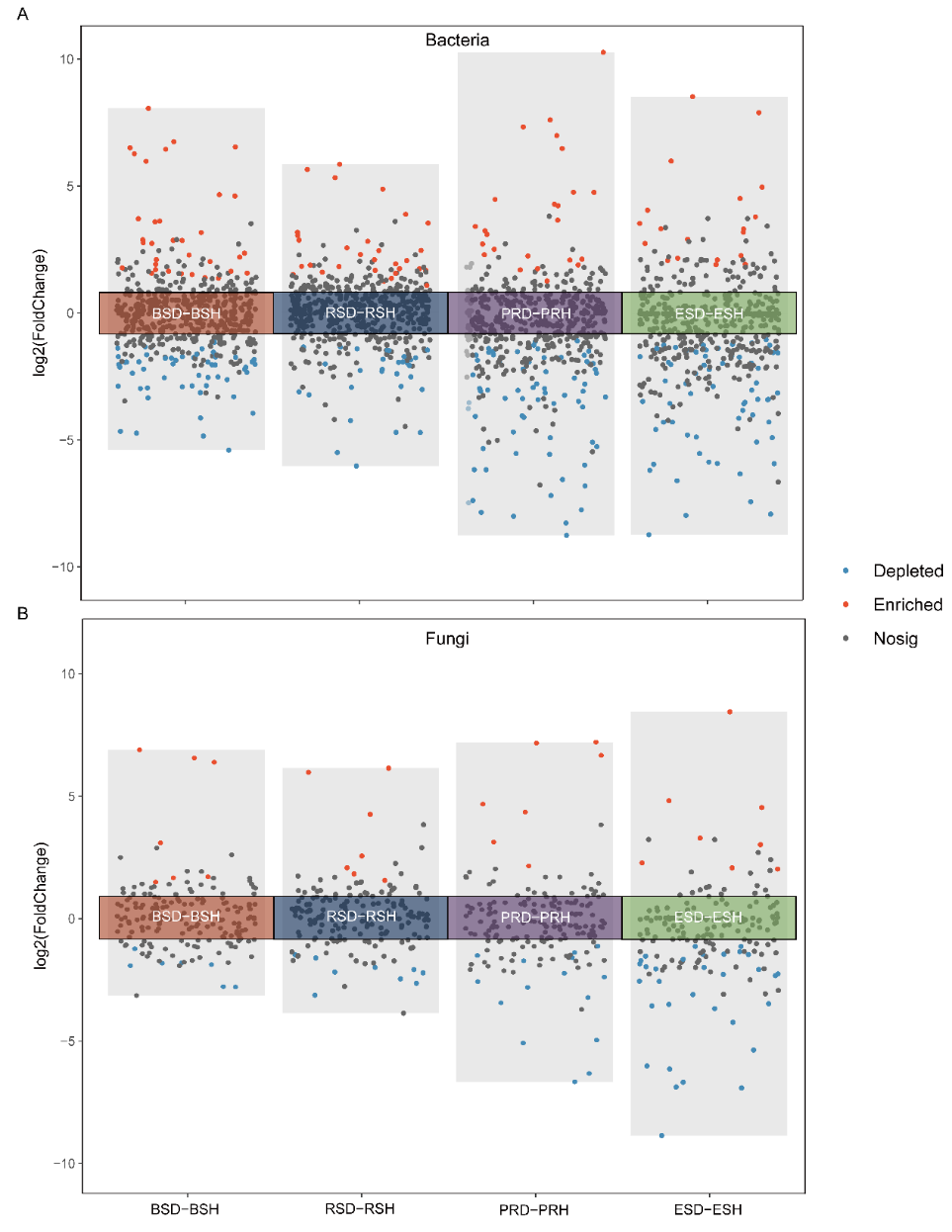


**Fig. S3** Volcano plots showing ASVs (relative >0.01%) enriched or depleted in the diseased compartment (bulk soil, rhizosphere, rhizoplane, and endosphere, respectively,) of the bacterial (A) and fungal community (B). BS, bulk soil; RS, rhizosphere; RP, rhizoplane; ES, endosphere; D, diseased tobacco plants; H; healthy tobacco plants.


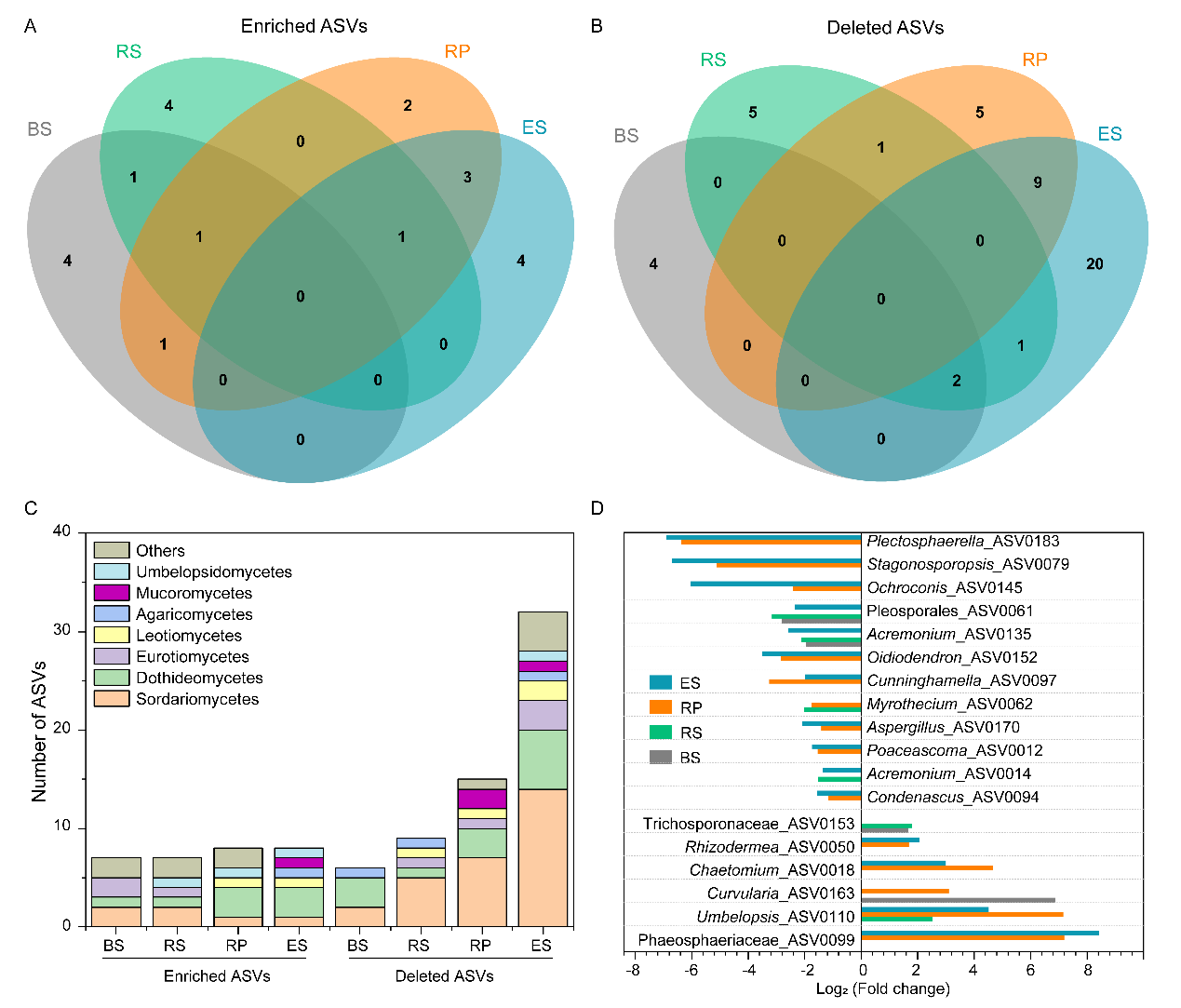


**Fig. S4** Taxonomic characteristics of differential fungi between healthy and diseased tobacco plants. Venn diagram showing the number of enriched (A) or depleted (B) ASVs in each compartment of diseased tobacco plants. Stacked bar chart indicated the taxonomy of enriched and deleted ASVs at the class level (C). (D) Shared enriched or depleted ASVs between the microbiomes of diseased bulk soil, rhizosphere, rhizoplane, and endosphere at the genus level (if not annotated to specific genera, using higher-level taxa to represent). BS, bulk soil; RS, rhizosphere; RP, rhizoplane; ES, endosphere.


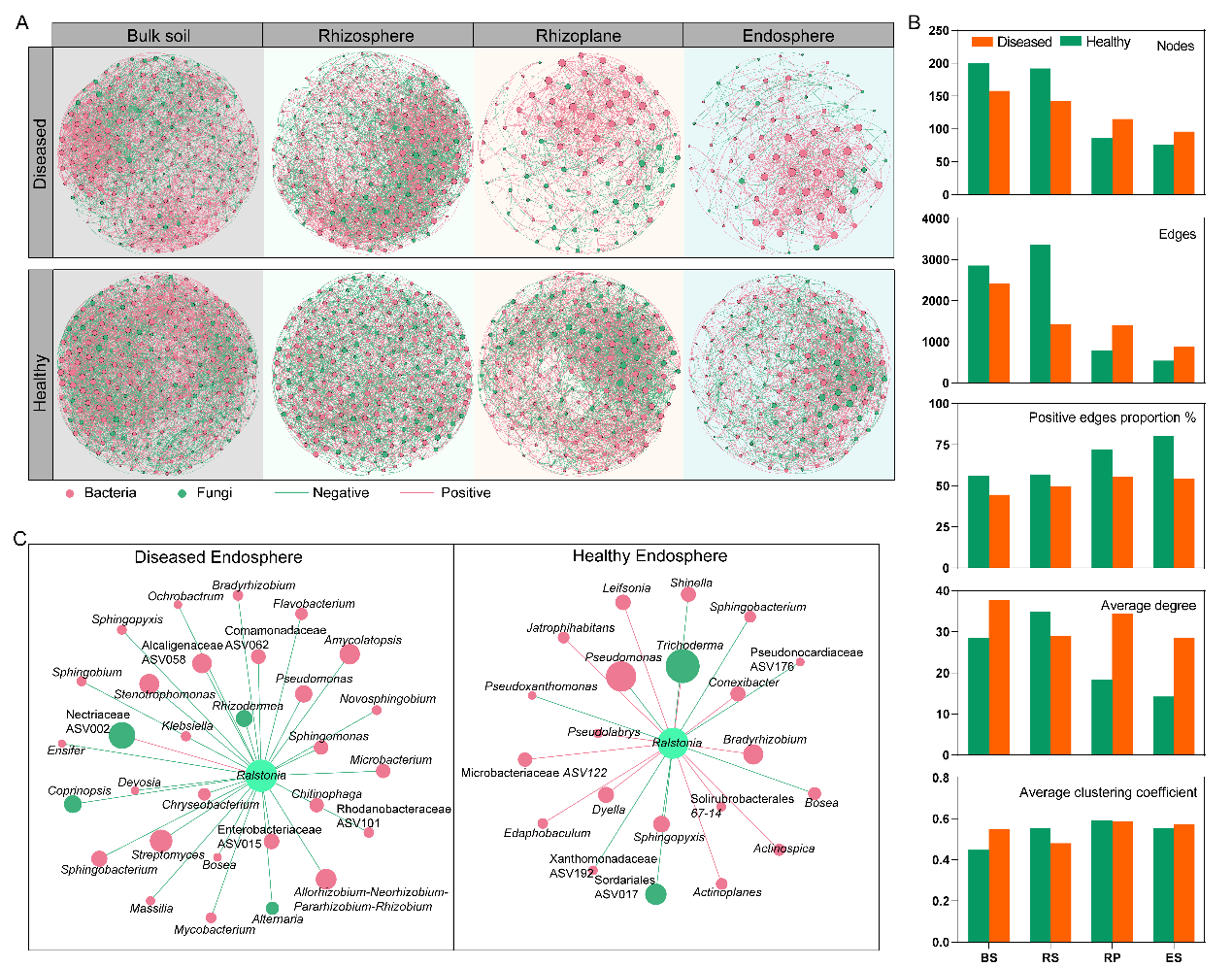


**Fig. S5** Pattens of the microbial co-occurrence networks between diseased and healthy tobacco plants across four compartments. A, Co-occurrence network analysis of diseased and healthy tobacco plants along the soil-root continuum. Nodes represent dominant bacterial and fungal genus (with relative abundances > 0.1%) that were shown in different colors based on the taxonomy of domain. The size of each node is proportional to the degree of genus. Lines in red and green denote positive and negative correlations, respectively. B, Network topological parameters for four niches bacterial networks. C, the correlations between *Ralstonia* and other genera in diseased and healthy endosphere.


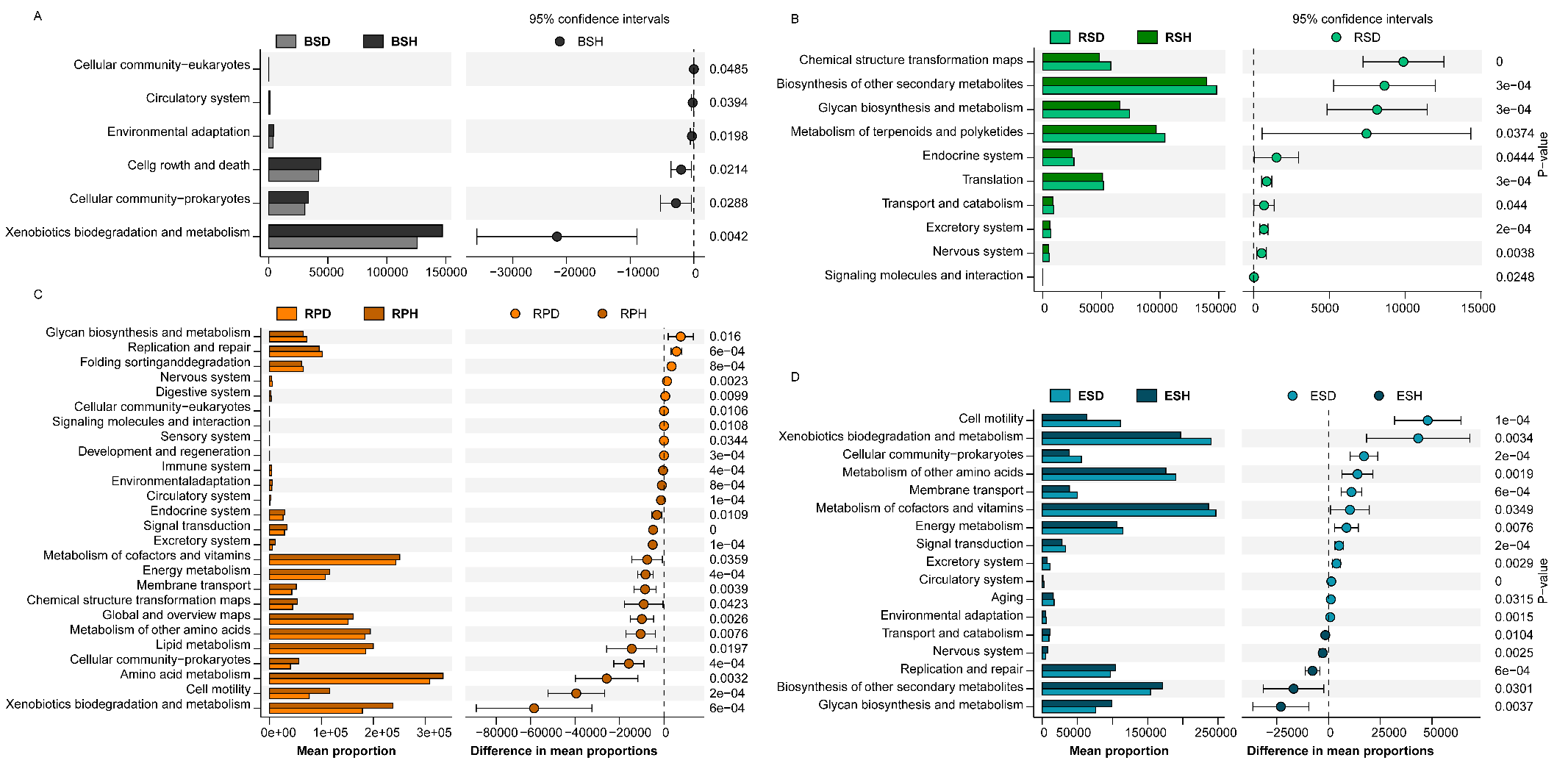


**Fig. S6** Difference in the abundance of bacterial functions between healthy and diseased bulk soils (A), rhizosphere (B), rhizoplane (C) or endosphere (D) at KEGG level 2. BS, bulk soils; RS, rhizosphere; RP, rhizoplane; ES, endosphere; D, diseased tobacco plants; H, healthy tobacco plants.


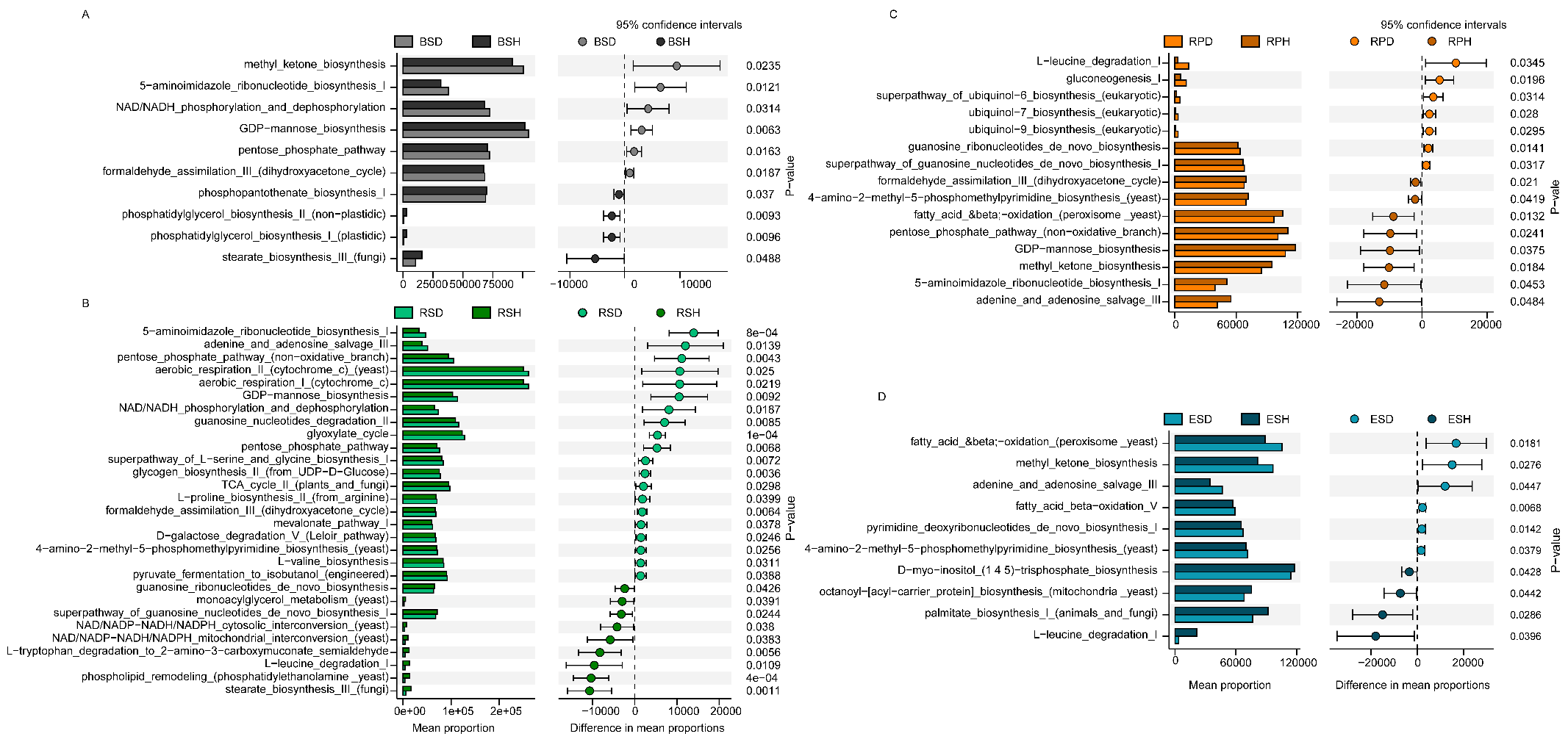


**Fig. S7** Difference in the abundance of fungal functions between healthy and diseased bulk soils (A), rhizosphere (B), rhizoplane (C) or endosphere (D). BS, bulk soils; RS, rhizosphere; RP, rhizoplane; ES, endosphere; D, diseased tobacco plants; H, healthy tobacco plants.
